# Coral-dwelling fish moderate bleaching susceptibility of coral hosts

**DOI:** 10.1371/journal.pone.0208545

**Published:** 2018-12-14

**Authors:** T. J. Chase, M. S. Pratchett, G. E. Frank, M. O. Hoogenboom

**Affiliations:** 1 Marine Biology and Aquaculture, College of Science and Engineering, James Cook University, Townsville QLD, Australia; 2 ARC Centre of Excellence for Coral Reef Studies, James Cook University, Townsville QLD, Australia; Department of Agriculture and Water Resources, AUSTRALIA

## Abstract

Global environmental change has the potential to disrupt well established species interactions, with impacts on nutrient cycling and ecosystem function. On coral reefs, fish living within the branches of coral colonies can promote coral performance, and it has been hypothesized that the enhanced water flow and nutrients provided by fish to corals could ameliorate coral bleaching. The aim of this study was to evaluate the influence of small, aggregating damselfish on the health of their host corals (physiology, recovery, and survival) before, during, and after a thermal-bleaching event. When comparing coral colonies with and without fish, those with resident fish exhibited higher *Symbiodinium* densities and chlorophyll in both field and experimentally-induced bleaching conditions, and higher protein concentrations in field colonies. Additionally, colonies with damselfish in aquaria exhibited both higher photosynthetic efficiency (F_V_/F_M_) during bleaching stress and post-bleaching recovery, compared to uninhabited colonies. These results demonstrate that symbiotic damselfishes, and the services they provide, translate into measureable impacts on coral tissue, and can influence coral bleaching susceptibility/resilience and recovery. By mediating how external abiotic stressors influence coral colony health, damselfish can affect the functional responses of these interspecific interactions in a warming ocean.

## Introduction

Coral reefs are among the most biodiverse and climate change vulnerable ecosystems [1,2], largely owing to the thermal sensitivity of habitat-forming scleractinian corals. Aside from causing widespread coral bleaching and coral loss [2,3], sustained and ongoing changes in environmental conditions may also threaten complex and critical interactions among coral reef organisms [2–5]. These complex interactions give rise to ecological processes that shape the structure and function of ecosystems, with feedbacks that are critical to reinforce or destabilize particular species-species and species-environment interactions [6–8]. For instance, aggregating damselfish and host corals are engaged in a positive feedback loop where symbiont damselfish increase coral growth, thereby increasing available habitat and attracting more damselfish [9]. Abnormally high ocean temperatures, however, disrupt the foundation interaction between the coral animal and its photosynthetic endosymbionts (*Symbiodinium* spp.), resulting in coral bleaching and mortality [1,2,10]. Severe bleaching events can lead to the loss of over 90% of local coral populations, especially in thermally-susceptible coral species, such as *Acropora*, *Pocillopora*, and *Stylophora*, [11–14], altering nearly all reef interactions and feedbacks dependent upon corals. Understanding the causes and impacts of bleaching on coral reef biodiversity and functioning requires knowledge of the environmental factors that stabilize or destabilize the core coral-*Symbiodinium* mutualism.

Coral symbioses are complex, multi-level networks of numerous species wherein the coral animal interacts with *Symbiodinium* with a complex microbial community [15], and with resident invertebrates and site-attached fish [16]. Various mechanisms act to stabilize or destabilize the coral holobiont. While temperature stress is often recognized as the primary driver of coral symbiosis breakdowns [1,9], other abiotic factors such as nutrient excess, changes in salinity, water flow, and light intensity [10] can also lead to bleaching, and mortality. Increased temperature also impacts symbiotic partners’ behavior and metabolism [17] as well as the host’s demands, leading to shifts in interactions from mutualisms to commensalism or parasitism, or abandonment of the symbiosis, or co-extinction [18].

Certain coral species, primarily branching corals from the genera *Acropora*, *Pocillopora*, *Seriatopora and Stylophora*, provide critical habitat for small aggregating fishes [19,20]. While these fish gain shelter, food, and refuge from coral colonies [20–22], they also provide benefits to corals. Certain fish species can enhance coral health by defending corals from predation [23], increasing nutrient concentrations in the water column [24–26], enhancing tissue aeration and increasing water flow between branches [27–29], slowing the progression of coral disease [30], and increasing overall growth [31–33]. Both increased nutrients (specificially altered nitrogen:phosphorous ratios) and water flow rates can moderate bleaching susceptibility (observed under field conditions) and the rates of recovery of bleached corals [34,35]. As coral-dwelling fishes can alter water flow and nutrient availability for corals, they can potentially influence coral resistance to bleaching and/or coral recovery from bleaching [36].

Multiple processes and feedbacks are likely to determine whether and how fish influence bleaching susceptibility and recovery of their host corals. Many damselfish species remain with their coral counterparts during and after thermal stress, even when corals are severely bleached [37,38]. As a result, the benefits that fish provide to corals can continue to operate during thermal stress conditions. Nutrient provision can lead to a proliferation of symbionts within coral tissue [31], and the nutrients excreted by fish living within coral branches might therefore prevent the collapse of the endosymbiotic algae population during temperature stress. Similarly, enhanced water flow can modulate mass-transfer rates and support gas exchange for photosynthesis; therefore, the swimming activity of fish living within coral branches might also stabilize symbiont population size and lessen the severity of bleaching [28,29,34]. However, bleaching can alter fish behavior, physiology and survival [39,40], and these changes potentially alter the nutrient provision and flow-moderation functions of fish living within corals [41]. Whether and how coral-associated fish aid corals in bleaching tolerance and recovery is unknown.

The objective of this study was to evaluate the influence of coral-dwelling fishes on the health of their host corals during and after thermal stress. We assessed the hypothesis that nutrient provision, aeration and water stirring by coral-dwelling fish act as ‘ecological buffers’ [42] that enhance coral health during temperature stress. Using a combination of field-based and aquarium experiments, this research aimed to elucidate the impacts of aggregating damselfish on: a) coral health under thermal bleaching conditions in the laboratory and in the field; and (b) coral health under ambient conditions in the field. Multiple physiological traits for the same coral fragments were measured to facilitate direct comparisons within colony bleaching treatments to assess whether fish ameliorate bleaching severity and/or enhance bleaching recovery.

## Materials and methods

### Ethics statement

All methods and experimental protocols were carried out in accordance with Great Barrier Reef Marine Park Authority permit (G15/37657.1), James Cook University Animal ethical guidelines and regulations (A2186), and James Cook University’s General Fisheries permit (170251). All coral and damselfish were returned to the site of collection (following JCU Ethics permit A2186), and select coral fragments (<8cm in length) were sacrificed for further laboratory tissue analysis, per GBRMPA permit G15/37657.1 None of the corals or damselfish collected were protected species. Data are available in S1, S2, S3, S4 and S5 Tables.

### Study system and location

An aquarium experiment and field observations were conducted to determine whether coral-dwelling damselfish enhance coral health before, during, and after thermal bleaching events. The symbiotic interaction between the coral-associated damselfish, *Dascyllus aruanus*, and its coral host was chosen due to the damselfish’s site fidelity [43], and its behavior of aggregating in social groups that remain close to the host coral, sleeping within the branches. *D*. *aruanus* is abundant within the Lizard Island lagoon [44] and is commonly found in groups of 2–10 fish on colonies of branching corals [19,24]. The coral *Pocillopora damicornis* was selected as a focal species for the aquarium experiment as it is a natural host of *D*. *aruanus* (and other damselfish species), is generally abundant on shallow coral reefs, and has often been used as a focal species in bleaching studies [44–46]. A different coral species, *Seriatopora hystrix*, was used in the field observations due to its local abundance and trajectory of bleaching at the time of field sampling. Both *P*. *damicornis* and *S*. *hystrix* are known to host damselfish, exist in a range of habitats with adult colonies similar in size ranges, and exhibit high bleaching susceptibilities [2,47]. Using previous literature on *S*. *hystrix* under natural conditions, in combination with *in situ* exposure to extreme temperatures similar to the aquarium experiment we conducted, provides a deeper understanding of fish impacts on corals during thermal stress. Source data on coral tissue and photosynthetic yield values for field and aquaria experiment are available in S1, S2, S3, S4 and S5 Tables.

Research was conducted at Lizard Island Research Station on the northern Great Barrier Reef (GBR), Australia (14°41’S, 145°27’E). An aquarium experiment investigating the effects of fish presence on coral bleaching severity and rates of recovery was conducted between June and August 2015, with all corals and fish used in these experiments collected from sites within the Lizard Island lagoon (Table 1). *In situ* bleaching observations were conducted in February and March of 2016, during the severe mass bleaching event [2]. Colonies of *S*. *hystrix* were tagged at four sheltered sites of the lagoon at depths between 0–2 m (n = 20 colonies per site, S1 Fig) and tracked for bleaching progression. These four sites had abundant small branching corals (mainly *S*. *hystrix*), both with and without target aggregating fish, and displayed bleaching during this timeframe. In contrast, during the observation period, other small branching corals with and without aggregating fish, located at deeper sites, had yet to exhibit signs of bleaching.

**Table 1:** Summary of the research objectives of this study, the general approach, and coral metrics used to investigate each objective.

Research Objective	General approach	Coral metrics analysed
*In situ observations of aggregating damselfish on coral hosts pre- and during bleaching conditions (in the field)*		
(i) Condition of *Pocillopora damicornis* with and without *Dascyllus aruanus* symbionts during non-bleaching conditions in the field	Colonies at one site within the Lizard Island lagoon	*Symbiodinium* density Total chlorophyll (α+ c) Total protein Tissue biomass
(ii) Condition of *Seriatopora hystrix* with and without *D*. *aruanus* symbionts during bleaching conditions in the field	Colonies at four sites within the Lizard Island lagoon	*Symbiodinium* density Total chlorophyll (α+ c) Total protein
*Impacts of aggregating damselfish on coral hosts under manipulative thermal bleaching experiment (in aquaria)*		
(iii) Condition of *P*. *damicornis* with and without *D*. *aruanus* symbionts during experimental bleaching temperatures in aquaria	Colonies under four experimental treatments: (i) ambient temp + colonies with fish; (ii) ambient temperature + colonies without fish; (iii) bleaching temperatures + colonies with fish; (iv) bleaching temperatures + colonies without fish.	*Symbiodinium* density Total chlorophyll (α+ c) Total protein Tissue biomass Photochemical efficiency (F_V_/F_M_)

### *In situ* observations pre- and during bleaching conditions

To confirm whether *D*. *aruanus* influenced the tissue composition of corals under ambient field conditions, fragments were sampled from small (20–50 cm diameter) *P*. *damicornis* colonies during non-bleaching conditions. In May of 2015, *P*. *damicornis* colonies with *D*. *aruanus* (n = 5, each with 2 to 10 damselfish) and without *D*. *aruanus* present (n = 4) were sampled within the Lizard Island lagoon between 0–4 m (similar depths per treatments). One fragment per colony was removed using a hammer and chisel. These fragments were analyzed for protein, symbiont density, total chlorophyll density, and tissue biomass (S4 Table) using the methods described below (see “Coral tissue analysis” below). Data were analyzed using a one-way analysis of variance (ANOVA) with fish presence as a factor. Statistical assumptions were assessed by analyzing residual plots, homogeneity of variance (Bartlett’s test), and normality (Shapiro-Wilks test).

To investigate the impacts of aggregating fish on corals during an *in-situ* bleaching event, 10 colonies were tagged at each of four sites (n = 40 colonies) within the Lizard Island lagoon in March 2016. At each site, *S*. *hystrix* colonies with *D*. *aruanus* (n = 5) and without *D*. *aruanus* (n = 5) were tagged, photographed, and sampled. *S*. *hystrix* was used, instead of *P*. *damicornis*, because it was more commonly found to host *D*. *aruanus* at these sites. One fragment from each colony was collected in March 2016 and analyzed for protein, symbiont density and total chlorophyll density (S5 Table). Coral colonies were checked 10 months post-tagging to quantify bleaching-related mortality under natural field conditions (see S1 Text and S1 Fig). To assess the impacts of fish on coral physiology (proteins, symbiont density, and total chlorophyll density) during *in situ* thermal bleaching, tissue composition data were analyzed using one-way analysis of variances (one-way ANOVAs) with Tukey’s HSD post-hoc tests (where applicable) using R statistical software. Statistical assumptions were assessed by analyzing residual plots, homogeneity of variance (Bartlett’s test), and normality (Shapiro-Wilks test).

### Manipulative thermal bleaching experiment

An aquarium experiment with a factorial design was established with ambient and heated water temperature treatments, and fish present versus absent. Corals were acclimated to aquarium conditions for two weeks prior to the start of the experiment. During this time any dead branches, algae and/or other invertebrates were removed. Ambient and heated sump tanks (1000 L, 2 sumps per temperature treatment) were established in a shaded outdoor area (daily maximum light intensity ~350 μmol photons m^-2^ s^-1^) with replicate aquaria positioned within each sump. Heated sump tanks each contained a 2400-watt water heater (TECO TK 1000 heaters, accuracy 0.1°C), and were equipped with 2–3 water pumps to ensure an even heat distribution. The two control (unheated) sumps received a supply of ambient seawater from the reef flat (23.5–25°C, dependent upon the time of day) for the entire duration of the experiment. The heated treatment was implemented in phases as follows: (i) Acclimation–corals were held at ambient temperatures for 7 days; (ii) Ramping—temperature was gradually raised from ambient to 32°C (typical of northern GBR summer temperatures, [2] over the course of 2 weeks (increase of~0.5°C day^-1^); (iii) Stress–corals were maintained at 32°C for 15 days, and; (iv) Recovery–temperature was decreased back to ambient over 8 days, and then maintained at ambient for 20 days to allow recovery. Spot-check temperature measurements were made for each tank multiple times daily using a handheld water-proof thermometer (±1°C accuracy, Dig-stem-1 Digital Thermometer, Instrument Choice AU). At the end of each of the acclimation, thermal stress, and recovery phases of the experiments, one fragment per colony (n = 114 in total) was sampled for subsequent quantification of tissue protein, symbiont density, total chlorophyll density, and tissue biomass.

Each individual aquarium (25 L volume) received an inflow of ambient seawater (~12 L hr^-1^) pumped directly from the Lizard Island lagoon, and was fitted with an air stone. This low flow rate of ~12 L hr^-1^ is representative of reef flow regimes, often ranging from 1 and 15 cms^-1^ [48]. Water from each aquarium flowed into the surrounding sump. This experimental set-up was designed to: a) ensure each replicate aquarium had an individual water supply so that fish-excreted nutrients did not contaminate tanks without fish, and b) ensure stable and equal water temperatures among replicate aquaria within each temperature treatment. Temperatures were maintained within ± 0.5°C of the desired level.

Replicate aquaria with fish and no-fish treatments were divided evenly between the sumps (10 replicates per sump). Each replicate had a small (~20–25 cm diameter) *P*. *damicornis* colony which was collected from the Lizard Island lagoon and which were naturally devoid of any resident fishes at the time of collection. Treatments with fish present contained six *D*. *aruanus* with a similar group biomass (individual fish biomass 0.5 to 5.6 g, group biomass 15 g ± 0.56) that were collected from the Lizard Island lagoon using a weak solution of clove oil [49,50] and hand nets. Damselfish were subject to a brief ‘freshwater rinse’ to remove any bacteria and parasites prior to being introduced to other fish and corals within each experimental treatment [51]. After 72 hours of acclimation, damselfish were weighed (wet weight, using a MS105 Semi-Micro Balance, Mettler Toledo, accuracy 0.001), measured (total length), and placed in aquaria with live *P*. *damicornis* colonies. Fish remained with the same conspecifics found in the field to maintain existing social groups and minimize aggressive behavior in aquaria. Fish number and biomass per aquarium were consistent with natural aggregations. Fish numbers and condition were inspected several times a day throughout the 66-day experimental period, particularly during feeding times when damselfish were actively moving in the water column. All corals and fish were fed multiple times a day to satiation [24] with enriched *Artemia salina* nauplii to supplement food naturally available in the seawater pumped from the nearby lagoon.

Linear mixed effects models with experimental phase, fish treatment and temperature treatment as factors, were used to assess whether fish presence affected each of the measured components of tissue composition during thermal stress using the function ‘lme’ in the package ‘nlme’ [52,53]. For all of these analyses, coral colony was included as a random effect to account for repeated measures of each colony at each phase of the experiment. Selected multiple comparisons (n = 12 post-hoc planned contrasts, see S6 Table) were performed using a model contrast matrix to determine: (a) whether the treatments differed immediately after acclimation, (b) effect of fish presence during bleaching, (c) effect of fish presence during recovery, and (d) long-term effect of fish presence two months after bleaching. Adjusted p-values and confidence intervals, to account for multiple contrasts, were utilized to determine which treatment combinations were significantly different from each other. Values in the text are specified as means ± standard error. All statistical analyses were performed using the R statistical software [52].

#### Photosynthetic efficiency as a proxy for bleaching severity

A Pulse Amplitude Modulated (PAM) fluorometer (Mini-PAM, Walz; for settings see S2 Text) was used to monitor the onset, severity, and recovery of coral bleaching nightly during the temperature stress, and every five days during acclimation and recovery, with three replicate measurements per colony per day. The dark-adapted F_V_/F_M_ (F_V_ is minimum fluorescence and F_M_ is maximum fluorescence)_,_ which is a measure of the maximum photochemical efficiency of symbionts present within coral tissue (e.g. [53], was measured approximately 2.5 hours after sunset (~21:00 h). F_V_/F_M_ was used as a proxy for coral bleaching severity as there is a relationship between the photosynthetic efficiency of symbionts (as measured using PAM fluorometry), symbiont density, and coral bleaching status [41,53–57]. Photosynthetic efficiency measurements were averaged per colony per night and the change in this metric over time was analyzed using piecewise regressions. This piecewise approach was used because the dynamics of F_V_/F_M_ differed during the different phases of the experiment. Linear regression was used to assess changes in F_V_/F_M_ for control (ambient temperature) corals throughout the experiment. For the colonies exposed to heat stress, linear regression was also used to assess changes in F_V_/F_M_ during recovery. Linear regressions were appropriate for analysis of F_V_/F_M_ during this phase of the experiment based on the distribution of the data. During heat stress, however, data from acclimation, ramping and thermal stress were analyzed using non-linear regression because changes in F_V_/F_M_ during these phases were strongly non-linear (S7 Table). A sigmoidal equation was chosen based on preliminary observation of the data [58], as:

$$Y=(mx+a)-(\frac{mx}{1+exp(-\frac{t-x\theta }{\omega })})$$ (1)

Where *Y* is the photosynthetic efficiency (F_V_/F_M_) on a given day during exposure to elevated temperature, *mx* is the maximum achievable efficiency, *a* is the minimum efficiency, *t* is time, *xθ* is the time at which *Y* is halfway between *mx* and *a*, and ω captures the rate at which efficiency declines. Because we were fitting different equations to the different sections of the data, we used a formal model selection process to determine which model best described the dynamics of F_V_/F_M_. Akaike’s Information Criterion (AIC) and subsequent weight (wAIC_i_) for each potential model (see S8 Table) were calculated [59,60]. The results presented are for equations fitted to the daily mean values for all colonies within each treatment. However, the model fitting was repeated for the data for individual colonies within treatments; that analysis yielded similar results with the same overall conclusions.

### Coral tissue analysis

In all three experiments (*in situ* natural conditions, *in situ* bleaching conditions, and *ex situ* thermal bleaching experiment) 1–2 coral fragments, approximately 6 cm in length, were collected from each colony. Fragments were subsequently frozen in liquid nitrogen during transport and maintained at -80°C prior to laboratory analysis. Tissue was removed from the skeleton using compressed air in 0.45 μm filtered seawater, collected, and homogenized. The resulting tissue suspensions were divided into aliquots for protein assays (1 ml), symbiont counts (0.9 ml with 0.1 ml of 10% formaldehyde, to preserve samples), total chlorophyll (5 ml), and tissue biomass (8 ml). Coral skeletons were retained to quantify fragment surface areas using the wax dipping technique [61]. Five coral colonies, all from the heated treatments in the manipulative thermal bleaching experiment (from colonies with and without fish), died during the recovery phase of the experiment. Tissue composition data for these dead corals were recorded as 0 for all metrics, to represent the biological consequences of coral death during bleaching events. Detailed raw data and methods of coral tissue analysis are provided in S3 Text and S2 Table.

## Results

### Effects of fish presence on corals before, during bleaching under natural conditions (*in situ*)

**Fig 1:**
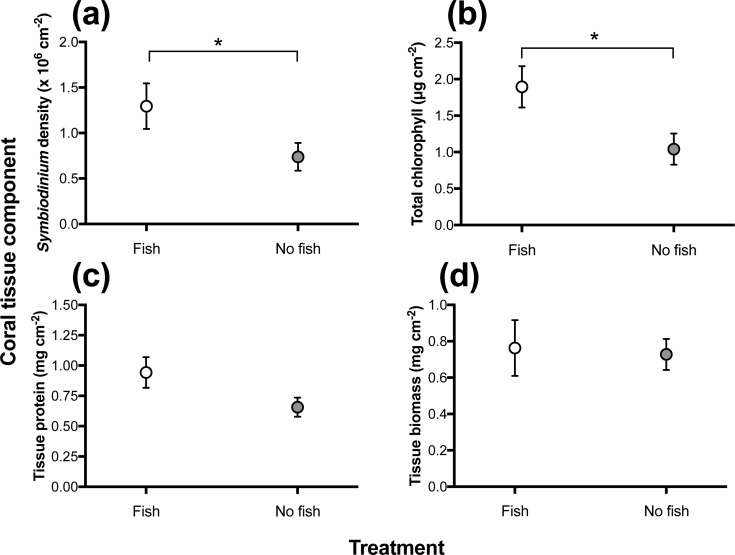
*In situ* levels of **(a)** endosymbionts (*Symbiodinium* density x10^6^ cm^-2^), **(b)** total chlorophyll (chl *a* + chl *c*, μg cm^*-*2^), **(c)** tissue protein (mg cm^-2^), and **(d)** tissue biomass (calculated via ash-free dry weight, mg cm^-2^) of naturally occurring *P*. *damicornis* colonies, with *D*. *aruanus* (n = 5) and without fish (n = 5) present. (*) denotes a significant difference between fish treatments, and *error bars* show S.E.

Under normal temperature conditions in the field, *P*. *damicornis* colonies with *D*. *aruanus* had significantly higher densities of *Symbiodinium* (ANOVA, F_1,8_ = 8.2, p = 0.02) and higher concentrations of total chlorophyll (ANOVA, F_1,8_ = 6.7, p = 0.03) than unoccupied colonies (Fig 1). In contrast, no significant differences were observed in protein concentration (ANOVA, F_1,8_ = 3.19 p = 0.112) or tissue biomass (ANOVA, F_1,8_ = 0.04 p = 0.85).

**Fig 2:**
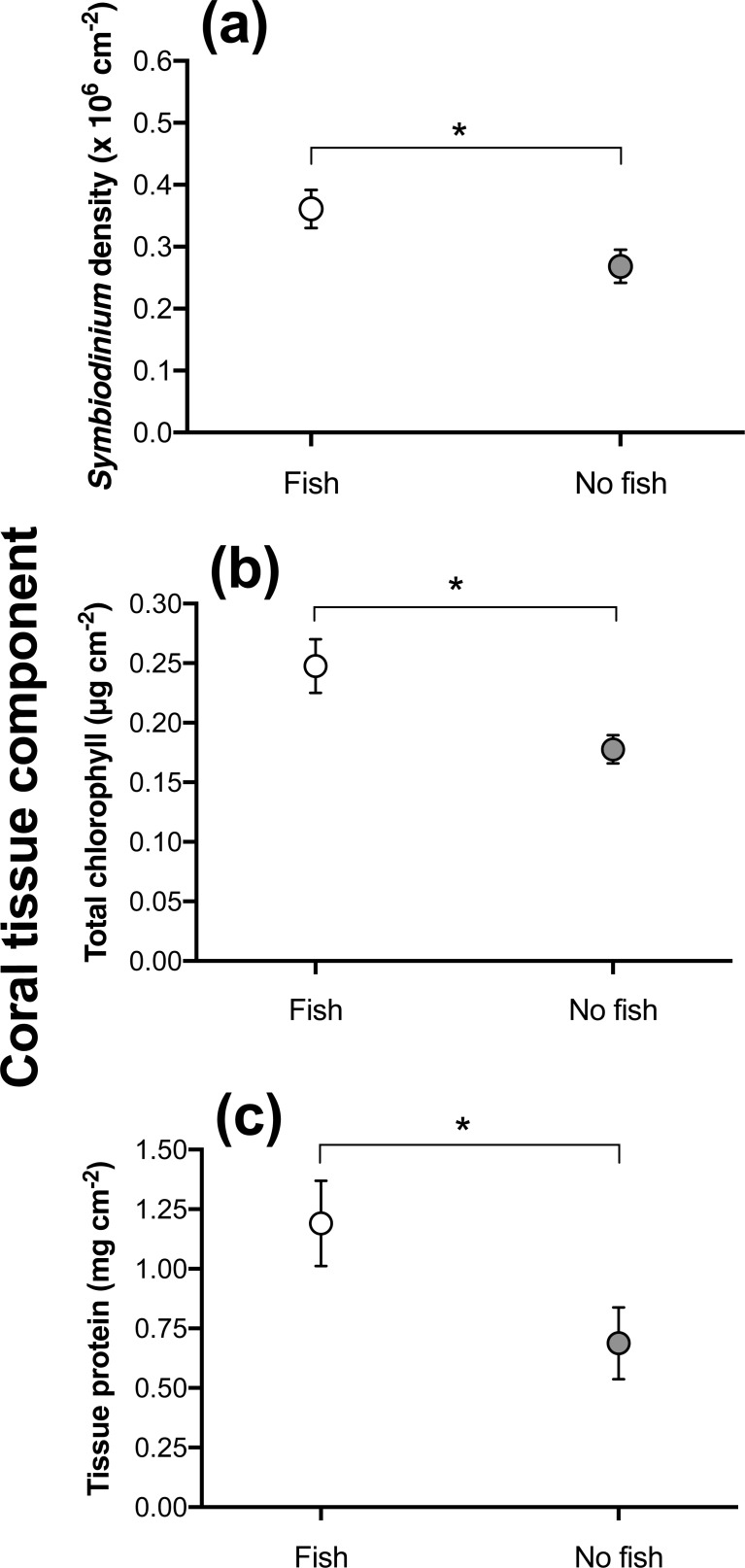
Differences in mean (±SE) levels of **(a)** endosymbionts (*Symbiodinium* density x10^6^ cm^-2^), **(b)** total chlorophyll (chl *a* + chl *c*, μg cm^*-*2^), and **(c)** tissue protein (mg cm^-2^) of naturally occurring *S*. *hystrix* colonies, with *D*. *aruanus* (n = 19) and without fish (n = 18) present during a coral bleaching event at Lizard Island. Colonies positioned at 1–3 m depth within four lagoonal sites with limited current activity. (*) denotes a significant difference between fish treatments, and *error bars* show S.E.

During the 2016 bleaching event at Lizard Island, *S*. *hystrix* colonies in the field were exposed to temperatures >33°C, which led to widespread bleaching and mortality. At the time of collection, *S*. *hystrix* colonies had an average of 0.32 x 10^6^ *Symbiodinium* cm^-1^ ± 0.02 (compared with typical ambient densities of 2.1 x 10^6^ *Symbiodinium* cm^-1^ ± 1.0 [47]). The effects of fish presence were consistent among sites for *Symbiodinium* density (ANOVA(treatment*site): F_3,30_ = 1.81, p = 0.17, Fig 2). Conjointly, average *Symbiodinium* densities were higher for colonies with fish than for colonies without fish (ANOVA treatment effect: F_1,33_ = 6.16, P = 0.018). In addition, average *Symbiodinium* densities differed between sites (ANOVA, site effect: F_3,33_ = 3.75, p = 0.02). No differences in total chlorophyll or proteins were detected among sites, however, both of the tissue variables depended upon fish presence (ANOVA: total chlorophyll, F_1,35_ = 7.29, p = 0.01, proteins: F_1,36_ = 4.50, p = 0.041, see Fig 2B and 2C). All colonies were monitored during the bleaching event and after a period of recovery of >6 months: in September 2016, >90% of colonies were dead and covered in filamentous algae regardless of fish presence/absence. Due to the severity of the bleaching event and the position of the colonies within a lagoon (higher recorded temperatures, see [62]), post-bleaching recovery was non-existent, resulting in widespread mortality of *S*. *hystrix* colonies (post-bleaching >90% of colonies were recorded as dead) and disappearance of symbiont damselfish.

### Effects of fish presence during experimental bleaching

**Fig 3:**
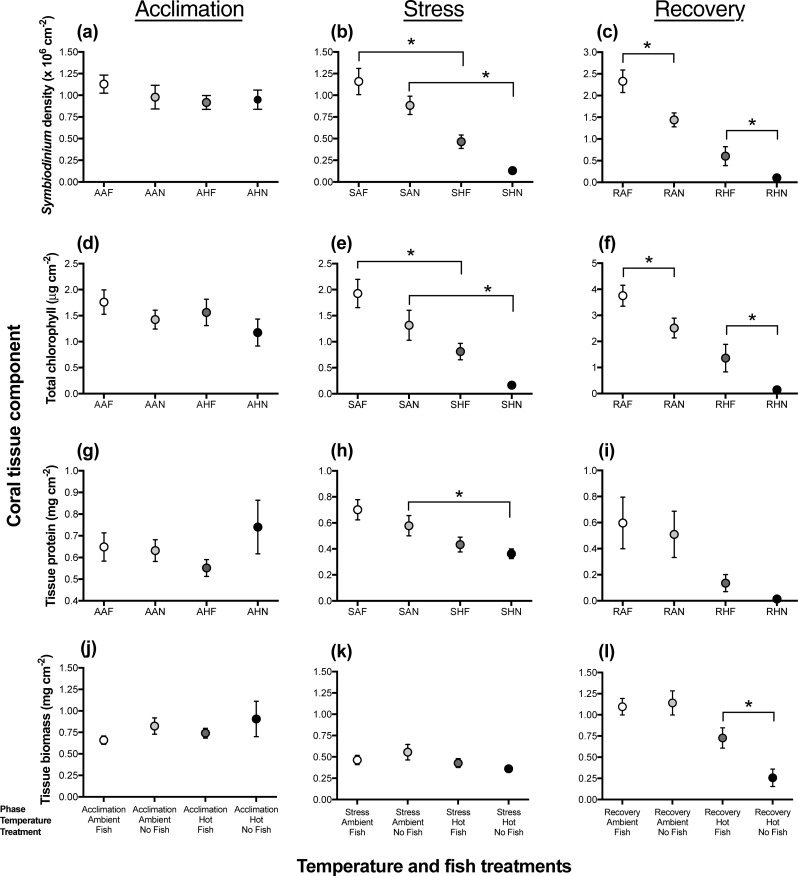
Levels of **(a-c)** endosymbionts (*Symbiodinium* density x10^6^ cm^-2^), **(d-f)** total chlorophyll (chl *a* + chl *c*, μg cm^-2^), **(g-i)** protein (mg cm^-2^), **(j-l)** tissue biomass (calculated via grams of ash-free dry weight mg cm^-2^) in experimental *P*. *damicornis* colonies, with *D*. *aruanus* for different temperature and fish treatments (ambient/fish: n = 9, ambient/no fish: n = 9, hot/fish: n = 10 and hot/no fish: n = 9) for three different experimental phases (Acclimation (25°C), Stress (temperature increased and held at 32°C for four weeks), and Recovery (temperature returned to 25°C). (*) denotes a significant difference between select comparisons fish treatments, and *error bars* show S.E. Refer to S6 Table for results of all 12 planned contrast per coral tissue components. Note difference in y-axis for panels C and F, to allow for visualization of variance between treatments. Data points per phase, temperature, and fish presence have been abbreviated to form 3 letter keys, as follows: A = acclimation, S = stress, R = recovery, A = ambient temperature, H = hot/bleaching temperature, F = fish present, N = fish absent. i.e. SHF = sample collected during stress phase of a hot temperature with fish present colony.

At the end of the acclimation phase during the manipulative thermal bleaching experiment, *Symbiodinium* density, chlorophyll density, protein concentration, and tissue biomass were approximately equivalent among all treatments (*in aquaria*: *Symbiodinium*: μ = 0.99 x 10^6^ *Symbiodinium* cm^-2^ ± 0.07; total chlorophyll: μ = 1.5 chl *a* + chl *c* μg cm^-2^ ± 0.10; protein: μ = 0.64 mg cm^-2^ ± 0.03; tissue biomass: μ = 7.8 mg cm^-2^ ± 0.048, see Fig 3A, 3D, 3G and 3J, Table 1, *planned comparisons* S6 Table). These values (see Fig 3A) were approximately the same as those for fragments sampled from the field (*in situ*: *Symbiodinium*: μ = 1.1 x 10^6^ ± 0.17 *Symbiodinium* cm^-2^; total chlorophyll: μ = 1.02 chl *a* + chl *c* μg cm^-2^ ± 0.15; protein: μ = 0.8 mg cm^-2^ ± 0.09; tissue biomass: μ = 7.5mg cm^-2^ ± 0.08, see Fig 1).

Due to the experimental design, temperature only differed between treatments in certain phases (e.g. in acclimation, all tanks received the same temperature). Consequently, *Symbiodinium* density only differed between treatments during the stress treatment and the recovery phase (significant phase*temperature treatment interaction, Table 2). During the stress phase, ambient colonies had significantly higher levels of *Symbiodinium* compared with their counterparts (*comparison*, SAF vs SHF: p = 0.001; SAN vs. SHN: p<0.001, Fig 3B) and this was observed in both the fish and no-fish treatments. All other planned contrasts for the Stress phase were non-significant (see S6 Table). After the recovery phase (Fig 3C), ambient colonies with fish had significantly higher *Symbiodinium* densities than colonies without fish (*comparison* RAF vs. RAN: p<0.001). After recovery, heated colonies with fish (including dead colonies with 0 *Symbiodinium* cm^-2^) had an average of 0.60 x 10^6^ ± 0.2 *Symbiodinium* cm^-2^, while heated colonies without fish had an average of 0.10 x 10^6^ ± 0.06 *Symbiodinium* cm^-2^ (*comparison* RHF vs RHN: p<0.021). Excluding dead corals, heated colonies with fish still had more *Symbiodinium* (0.67 x 10^6^ ± 0.23 *Symbiodinium* cm^-2^) than heated colonies without fish (0.19 x 10^6^ ± 0.09 *Symbiodinium* cm^-2^). Between the stress and recovery phases (~30 days), *Symbiodinium* in heated colonies with fish increased (+0.14 x 10^6^ *Symbiodinium* cm^-2^), while *Symbiodinium* in heated colonies without fish decreased slightly (-0.03 x 10^6^ *Symbiodinium* cm^-2^). Declines in F_V_/F_M_ below 0.7 were associated with declines in *Symbiodinium* concentrations from 1 x 10^6^ cells per cm^2^ to <0.2 x 10^6^ cells per cm^2^ (S2 Fig).

**Table 2:** Linear mixed effect model of the effect of phase, temperature, and fish presence (*D*. *aruanus*) on experimental *P*. *damicornis* colonies for (i) *Symbiodinium* density, (ii) total chlorophyll density, (iii) total proteins (iv) and tissue biomass (as part of the manipulative thermal bleaching experiment), where coral colony was included as a random effect.

Coral component and factor	Df	*F*	*P*
*Symbiodinium*			
Phase	2,66	13.6610	<0.001
Temperature	1,33	73.0350	<0.001
Treatment	1,33	14.5070	<0.001
Phase:Temperature	2,66	30.2860	<0.001
Phase:Treatment	2,66	6.2300	<0.001
Temperature:Treatment	1,33	0.8580	0.360
Phase:Temperature:Treatment	2,66	0.7610	0.470
*Total Chlorophyll*			
Phase	2,69	10.683	<0.001
Temperature	1,41	49.310	<0.001
Treatment	1,41	17.059	<0.001
Phase:Temperature	2,69	18.651	<0.001
Phase:Treatment	2,69	3.4260	0.038
Temperature:Treatment	1,33	0.1260	0.730
Phase:Temperature:Treatment	2,69	0.0980	0.910
*Protein*			
Phase	2,66	12.7377	<0.001
Temperature	1,33	16.1734	<0.001
Treatment	1,33	0.4165	0.523
Phase:Temperature	2,66	6.7671	<0.001
Phase:Treatment	2,66	1.3440	0.268
Temperature:Treatment	1,33	0.4041	0.529
Phase:Temperature:Treatment	2,66	0.4201	0.659
*Tissue biomass*			
Phase	2,126	15.9175	<0.001
Temperature	1,126	12.3097	<0.001
Treatment	1,126	0.0002	0.988
Phase:Temperature	2,126	11.3356	<0.001
Phase:Treatment	2,126	2.7551	0.067
Temperature:Treatment	1,126	2.8269	0.095
Phase:Temperature:Treatment	2,126	1.1974	0.308

Similar to *Symbiodinium* densities, the presence of fish had a significant effect on total chlorophyll density in the interactions between phase, temperature, and treatment (Table 2) within the manipulative thermal bleaching experiment. During the stress phase, ambient temperature colonies had significantly higher levels of chlorophyll when compared with their heated/bleaching counterparts (comparison, SAF vs SHF: p = 0.008; SAN vs. SHN: p = 0.007, Fig 3E). Additionally, during stress, heated colonies with fish had an average of 0.67 μg cm^-2^ chlorophyll more than heated colonies without fish. During the recovery phase (Fig 3E and 3F), colonies with fish had significantly higher levels of chlorophyll density than colonies without fish (comparison RAF vs. RAN: p<0.002, RHF vs RHN: p = 0.005). All other planned comparisons for the Stress phase were non-significant. Analysis further indicated that between stress and recovery phases, total chlorophyll in heated with fish increased greatly (+0.52 μg cm^-2^ chlorophyll), while total chlorophyll in heated colonies without fish only increased slightly (+0.04 μg chlorophyll cm^-2^). Excluding dead corals, heated colonies with fish still had significantly more chlorophyll (1.49 ± 0.53 μg chlorophyll cm^-2^) than heated colonies without fish (0.127 ± 0.12 μg chlorophyll cm^-2^).

While there were no effects of fish presence on tissue protein concentrations or tissue biomass, differences between temperature treatments were evident (Table 2 and Fig 3G, 3H, 3I, 3J, 3K and 3L). Overall, colonies with fish exhibited slightly higher values of protein and tissue biomass than colonies without fish, in both stress and recovery phases. During the stress phase, heated corals contained ~2x less protein than ambient temperature colonies; ambient colonies with fish had 0.27 mg cm^-2^ more protein than stress heated colonies with fish (comparision SAF vs SHF p = 0.046). Additionally, during the stress phase, ambient colonies without fish had 0.22 mg cm^-2^ more protein than stress heated corals without fish. These relationships were exaggerated in the recovery phase with ambient corals having ~4 times more protein than heated corals (Fig 3I). For tissue biomass, during recovery phase (Fig 3L), heated colonies with fish increased in biomass (+0.299 mg cm^-2^), while biomass in heated colonies without fish decreased (-0.1 mg cm^-2^); these colonies with fish had significantly higher levels of chlorophyll density than colonies without fish (*planned comparison* RHF vs RHN: p<0.012).

#### Change in photosynthetic efficiency during and after manipulated temperature stress

**Fig 4:**
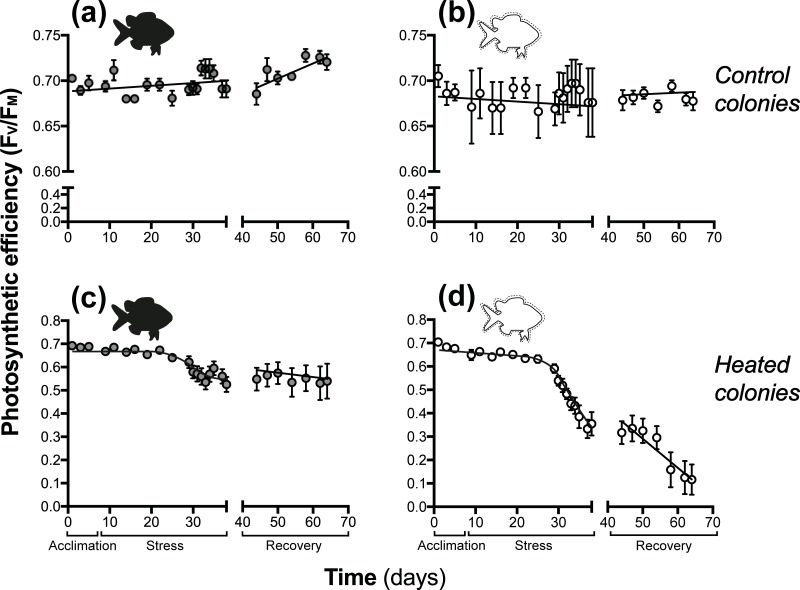
Temporal changes in photosynthetic efficiency (F_V_/F_M_) of *P*. *damicornis* with (**a** and **c**) and without *D*. *aruanus* (**b** and **d**) under control (**a** and **b**) and heated (**c** and **d**) treatments. Data are presented for all phases of the experiment: Acclimation (days 1–7), Temperature Stress (days 8–37) and Recovery (days 38–66); and points and error bars show means and S.E. for n = 9 colonies per treatment group. Solid lines show best fit regression lines (for line equations regression coefficients see Table 3). Black fish symbols represent colonies with fish, and white symbols represent colonies without fish. Note different y-axis ranges were used for visual clarity of effects.

Prior to the temperature stress (during acclimation) in the manipulative thermal bleaching experiment, all colonies of *P*. *damicornis* had approximately equivalent photosynthetic efficiency (F_V_/F_M_ = ~0.7). The best model to explain inter-colony differences in photosynthetic efficiency through the course of the experiment included both temperature treatment and fish treatment (Fig 4A; Table 3, wAIC for the model which fitted separate responses for all treatments = 1.0). For colonies with fish and subject to ambient conditions, F_V_/F_M_ increased gradually over time, while colonies subjected to ambient temperature without fish had constant F_V_/F_M_ throughout the entire experiment (Table 3 and Fig 4A and 4B). Overall, ambient corals with fish exhibited slightly higher and more consistent values of F_V_/F_M_ compared with colonies without fish (Fig 4B). Irrespective of fish presence, F_V_/F_M_ decreased in heated corals during the stress phase, when temperatures exceeded 30°C, typical of natural bleaching events at Lizard Island (Table 3 and Fig 4C and 4F). However, heated colonies without fish exhibited a more pronounced decline in F_V_/F_M_ to less than half of its initial value (0.7 to ~0.3) when compared with a 30% decrease observed in heated colonies without fish (0.7 to ~0.5). The parameters describing the non-linear relationships between F_V_/F_M_ and time during the experiment (*mx, xθ*, ω, and α) depended upon temperature treatment and fish presence (Table 3). During recovery, heated colonies with fish continued to experience a very slight decrease in F_V_/F_M_ (Fig 4C and Table 3) for the duration of the experiment. However, F_V_/F_M_ in heated colonies without fish continued to decline (Fig 4D and Table 3) with an average F_V_/F_M_ of close to 0.25 at the end of the experiment. Differences in photosynthetic function were correlated to an increased density of *Symbiodinium* (S3 Fig).

**Table 3:** Comparison of regression models testing the effects of temperature (ambient: 25°C or hot: 32°C) and fish presence (fish or no fish) on *P*. *damicornis* photosynthetic efficiency (F_V_/F_M_), fitting the data through the means for colonies within treatments for the Acclimation and Stress experimental periods during the manipulative thermal bleaching experiment. Akaike’s information criteria (AIC) and AIC differences (ΔAIC) were calculated per model selection practice [58–60]. See S8 Table for calculations with individual points yielding similar results as mean models (mean model results presented here).

**No.**	Model	N	AIC	delta AIC	wAIC
1	All data	76	-170.44	241.32	0.00
2	By temperature treatment	76	-331.45	80.31	0.00
3	By fish treatment	76	-181.39	230.37	0.00
4	By temperature treatment by fish treatment	76	-411.76	0.00	1.00

## Discussion

This study demonstrates that coral-dwelling fishes may reduce bleaching severity, as well as enhance post-bleaching recovery, for host corals. Using a combination of field-based observations and aquarium experiments, we show that corals that host fishes have higher *Symbiodinium* densities and chlorophyll concentration when compared to colonies without resident fishes. When subjected to thermal anomalies, corals hosting fishes continued to have higher *Symbiodinium*, chlorophyll, and tissue protein. The mechanisms underlying these findings are likely to include inputs of nutrients from fish excretion, and aeration and water stirring from fish swimming within branches, that moderate the effects of thermal stress. However, under severe warming conditions, >90% bleached corals died regardless of the presence or absence of resident fishes.

Beneficial effects of fishes on *Symbiodinium* densities and chlorophyll concentrations of host corals have been recorded previously [26,63,64]. In this study, we observed that colonies maintained in aquaria for 66-days with fish had almost two-fold higher *Symbiodinium* and chlorophyll levels than colonies without fish. The elevated levels of *Symbiodinium* and chlorophyll translate into higher photosynthesis rates (29), and faster overall growth rates in colonies with aggregating damselfish [33,64]. While differences in photosynthetic function were directly related to an increased density of *Symbiodinium*, additional physical components and processes associated with fish presence, such as increased net oxygen exchange and reduction of the diffusive boundary layer [28] due to water stirring and other specific behaviors of resident fishes, may also explain variations in photosynthetic function.

The benefits that fish can provide to corals have been identified in at least seven fish families [24,65–67]. However, benefits to host corals are best understood for damselfishes (family Pomacentridae) that exhibit some of the highest levels of association with small branching corals [20]. At the level of the coral population, these benefits for coral health are likely substantial, as aggregating damselfishes are widely distributed across the Indo-Pacific, are present in nearly all reef zones and, in certain habitats, more than 80% of branching corals are engaged in Pomacentrid-coral associations [19,24]. Consequently, resident aggregating fish potentially play an important role in buffering coral populations from certain environmental changes.

Higher baseline levels of *Symbiodinium* and chlorophyll in the field due to fish presence may counteract high energy requirements of bleaching before expulsion and coral starvation [68]. The smaller decrease in F_V_/F_M_ of colonies with fish is consistent with a ~22% increase in photosynthesis due to fish ventilation observed in a previous study [29]. This continual ventilation of the colony interior could reduce holobiont stress during bleaching by enhancing photosynthetic gas exchange and ameliorating oxidative stress. Comparable to other studies, photosynthetic efficiency values (especially in corals without fish) were still considerably low 4 weeks post-bleaching; marked decreases in bleached colonies of *P*. *damicornis* were reported during the 1998 bleaching event at Heron Island, GBR [47,54], where *P*. *damicornis* colonies F_V_/F_M_ values dropped >25% from ~0.60 to 0.45, similar to this experiment.

Similar to ambient conditions [25,33,64], fish services continue to enhance coral health under bleaching conditions, as examined in this study. These small-scale feedbacks (i.e. services between damselfish and corals) influence colony physiology and can accumulate to influence the stability and resilience of coral populations at larger scales [69]. By increasing functioning in a pre-disturbance state, there is evidence that corals with fish can temporarily experience continued benefits during certain disturbances, along with expedited recovery. However, these benefits require that fish remain with their host colonies during and after disturbance. In the case of bleaching, abandonment of the colony by resident damselfish has been documented only after the coral died and succumbed to algae overgrowth [38], but not during the states of declining coral health [70]. In this case, *D*. *aruanus* is able to maintain swimming performance at high temperatures, [71,72] supporting the idea that this species of fish can maintain fish-derived services to host corals (remaining with the colony and swimming within branches, see [38]), as observed in this study.

Regardless of fish, these *S*. *hystrix* colonies still bleached severely and displayed approximately two-fold lower values of *Symbiodinium* compared with those observed under non-bleaching conditions [47]. The intensity and duration of the bleaching may overwhelm natural resilience limits [73,74], and result in a loss of advantageous fish services, resulting in severe bleaching and mortality (>90% whole colony mortality) for field colonies. This is consistent with widespread bleaching events, leading to high coral mortality resulting in short-term changes such as loss of suitable habitat for aggregating fish and long-term changes such as loss of complexity and rise of alga-dominated states [14].

The benefits accrued to host coral colonies from hosting high abundance or biomass of resident fishes is strongly context-dependent [75]. Most notably, benefits of reef fishes on host corals are most apparent under low-flow conditions [24], potentially due to greater capacity for nutrient enrichment, due to increased residency time of water within the host coral colony [64]. Similarly, the positive effects of fish on host corals were generally apparent in aquaria settings, but not in the field. In aquaria, the presence of coral-dwelling fishes resulted in higher survival and partial recovery of coral colonies. It is likely that close interactions between fish and corals, restricted by aquaria space, enhanced effects of fish on corals during temperature stress. Additionally, controlled factors in aquaria, such as high food levels, low flow levels, low light stress, and removal of other external factors (i.e. coral predators) may not fully simulate *in situ* conditions and may limit comparison to natural field conditions. Nutrient pollution is an increasing global stressor and can result in localized direct effects on corals [26,76]. Further research is needed to assess whether the nutrient subsidy via fish may continue to produce positive effects for corals, have a negative additive effect with high ambient nitrogen levels (24), or neutralize certain fish services.

### Conclusions

Global climate change, and especially ocean warming, is greatly altering the structure of coral reef assemblages [17,77,78], with concomitant effects on species interactions and ecosystem function. In this study, the critical symbiotic association between corals and zooxanthellae (*Symbiodinium*) is moderated by the presence and behaviour of coral-dwelling damselfishes. Under certain conditions, the presence of these fishes may actually reduce vulnerability to coral bleaching, thereby ensuring persistence of host corals [8]. In this study, this feedback was relatively weak, and did not prevent host coral bleaching nor loss during severe thermal stress in the field. However, increased densities of coral-dwelling fishes or stronger associations between fishes and corals may confer increased resilience [8,79], thereby buffering the effects of global environmental change.

## Supporting information

S1 Text Aquaria experimental bleaching field recovery. (DOCX)

S2 Text Photosynthetic yield PAM settings. Mini pulse-amplitude modulator (MINI-PAM), Heinz Walz GmbH Germany, settings as used for all F_V_/F_M_ and rapid light curve (RLCs) measurements.
(DOCX)

S3 Text Coral tissue analysis. (DOCX)

S1 Table Raw data: Mean photosynthetic yield (F_V_/F_M_) for *Pocillopora damicornis* colonies in aquaria bleaching experiment at Lizard Island Research Station during Acclimation and Stress time periods. (PDF)

S2 Table Raw data: Mean photosynthetic yield (F_V_/F_M_) for *Pocillopora damicornis* colonies in aquaria bleaching experiment at Lizard Island Research Station during the Recovery period. (PDF)

S3 Table Raw data: Coral tissue compositions for *Pocillopora damicornis* colonies in aquaria bleaching experiment at Lizard Island Research Station. (PDF)

S4 Table Raw data: Coral tissue compositions for *in situ Pocillopora damicornis* colonies around Lizard Island, under non-bleaching conditions. (PDF)

S5 Table Raw data: Coral tissue compositions for *in situ Seriatopora hystrix* colonies around Lizard Island, during a bleaching event. (PDF)

S6 Table Summary of results of multiple selected comparisons (n = 12) as a post hoc test for the linear mixed effects model of the effects of phase, temperature, and fish presence (*D. aruanus*) on *P. damicornis* colonies. Each of the 12 comparisons are completed for four coral tissue parameters: *Symbiodinium*, total chlorophyll, protein, and tissue biomass. For each comparison, the upper and lower confidence intervals and adjusted p-value is listed.
(DOCX)

S7 Table Comparison of linear (mx, b) and non-linear (mx, x0, w, a) regression equation and coefficients for photosynthetic efficiency (F_V_/F_M_) during Acclimation/Stress phase and Recovery phase for coral colonies under ambient and heated temperatures and with and without fish treatments. (DOCX)

S8 Table Comparison of regression models testing the effects of temperature (ambient: 25°C or hot: 32°C) and fish presence (fish or no fish) on *P. damicornis* photosynthetic efficiency (F_V_/F_M_) through fitting the data points for each individual colony within treatments for F_V_/F_M_ associated with Acclimation and Stress experimental periods. Akaike’s information criteria (AIC) and AIC differences (ΔAIC) were calculated per model selection practice of Burnham and Anderson (2002) and Hoogenboom et al. (2011). Constructing the model with means (mean models presented in results), allows for regressions to explain a greater amount of variation in the data, compared with using all the individual points, but reduced statistical power. Data fitted through individual points yield similar results as mean models.
(DOCX)

S1 Fig Location of four *in situ* bleaching colonies (*S. hystrix*) within the Lizard Island Lagoon. (DOCX)

S2 Fig Differences in photosynthetic efficiency (F_V_/F_M_) of *P. damicornis* corals returned to the field, six months post aquaria bleaching experiment. F_V_/F_M_ values were recorded post aquaria bleaching experiment, February 2016, when GBR: (a) F_V_/F_M_ of coral colonies under new fish categories due to movement and additional fish species present, irrespective of past experimental treatments of heat and fish presence. New fish category includes aggregating fish (*D*. *aruanus*, *D*. *reticulatus*, *P*. *amboinensis*, and *P*. *moluccensis)* present during multiple observations. No fish SE = 0.0170, and Any fish SE = 0.0087. (b) F_V_/F_M_ of coral colonies under category of only *D*. *aruanus* still present. *D*. *aruanus* absent SE = 0.0099, and *D*. *aruanus* present SE = 0.0126. (*) denotes a significant difference between fish treatments and *error bars* show SE. One-way analysis of variance (ANOVA) were performed on PAM data, 6-months post-experiment test for differences in F_V_/F_M_ levels in field samples of *P*. *damicornis*. Data for F_V_/F_M_ analysis met assumptions of normality (Shapiro-Wilks test) and homogeneity of variance (Bartlett’s test).
(DOCX)

S3 Fig Relationship between symbionts (*Symbiodinium* density x10^6^ / cm^2^) and photosynthetic efficiency (F_V_/F_M_ of *P. damicornis* colonies at three different time periods in aquaria experiment. Photosynthetic efficiency (F_V_/F_M_) of *P*. *damicornis* colonies was recorded during: Acclimation (day 5), Stress (day 37) and Recovery (Day 66), in aquaria experiment. Linear regression analysis (Pearson’s correlation r^2^ = 0.5468, F_1,10_ = 12.07, p = 0.0060, y = 0.2266x + 0.378) suggests direct correlation between *Symbiodinium* and photosynthetic efficiency in experimental corals.
(DOCX)
