# Supplementary material for: Coral-dwelling fish moderate bleaching susceptibility of coral hosts
Source: PLoS One. 2018 Dec 14;13(12):e0208545. doi: 10.1371/journal.pone.0208545 (PMC6294555; doi:10.1371/journal.pone.0208545)
Supplement: S1 Text — (DOCX) [file pone.0208545.s001.docx]

**S1 Text:** Aquaria experimental bleaching field recovery.

*The following supplement accompanies the article*

Coral-dwelling fish moderate bleaching susceptibility of coral hosts

**List of authors**

TJ Chase^1,2^*, MS Pratchett^2^, GE Frank^1^, and MO Hoogenboom^1, 2^

___________________________________________________________________________

**S1 Text:** *Aquaria experimental bleaching field recovery*

Fish and colonies were monitored for 1 week upon being returned to the field and six-months post-experiment. There was a rapid decline of fish on experimentally occupied colonies that were places 1-2m off the surrounding patch-reefs, due to predation (via *P. leopardus*, personal observation) and movement to adjacent healthy corals. Rapid decline of fish density on these manipulated coral patches following fish relocation is common due to short-term processes and adjustment to novel habitat features (1). At six months post experiment 45% of corals ‘experimentally occupied corals’ were still occupied with *D. aruanus* (including newly settled recruits). Out of all the experimental colonies, irrespective of bleaching status or previous fish treatment, over 72% were occupied by *D. aruanus* or additional damselfish from the surrounding area (mainly, *P. ambionensis* and *P. moluccensis*). At six-months post experiment, February 2016, experimental *P. damicornis* corals were already subject to bleaching conditions in the field. As significant resident fish shuffling, displacement, and recruitment has occurred over six months, original fish treatment categories were confounded. Of the original experimental colonies, 40% exhibited mortality, covered in filamenous algae in February 2016; this could be due to delayed effects of experimental treatment, increases in mortality commonly observed in dislodged corals (2), or onset of field beaching. Of these dead colonies, half contained small resident damselfish. 84% of still alive experimental colonies were inhabited by fish, 43% of which had *D. aruanus* present. Using new fish treatment categories based on fish position in February 2016, alive *P. damicornis* colonies with fish present displayed higher mean F_V_/F_M_ values (based on 6 replicates over two non-sequential nights) than colonies without fish, (ANOVA: F_1,154_ = 0.0686, p = 0.0079). When regrouping the colonies into with only *D. aruanus* present and no *D. arunaus* present (irrespective of other fish present), colonies with *D. aruanus* also displayed higher mean F_V_/F_M_ values (ANOVA: F_1,154_ = 0.05175, p = 0.0215, Fig 6.). Here, the difference in photosynthetic efficiency, cannot be solely attributed to fish presence, as previous occupation, experimental temperature treatments, and current bleaching onset could potentially confound results.

**References:**

1. Lassig B (1976) Filed observations on the reproductive behavior of Paragobiodon spp. (Osteichtyes: Gobiidae) at Heron Island, Great Barrier Reef. Marine Behavioral Physiology 3:283-293
2. Ward S (1993) The effect of damage on the growth, reproduction, and storage of lipids in the scleractinian coral, *Pocillopora damicornis* (Linnaeus) Journal of Experimental Marine Biology and Ecology 187: 193-206
