# Supplementary material for: Coral-dwelling fish moderate bleaching susceptibility of coral hosts
Source: PLoS One. 2018 Dec 14;13(12):e0208545. doi: 10.1371/journal.pone.0208545 (PMC6294555; doi:10.1371/journal.pone.0208545)
Supplement: S2 Text — Mini pulse-amplitude modulator (MINI-PAM), Heinz Walz GmbH Germany, settings as used for all FV/FM and rapid light curve (RLCs) measurements. (DOCX) [file pone.0208545.s002.docx]

**S2 Text:** PAM settings

*The following supplement accompanies the article*

Coral-dwelling fish moderate bleaching susceptibility of coral hosts

**List of authors**

TJ Chase^1,2^*, MS Pratchett^2^, GE Frank^1^, and MO Hoogenboom^1, 2^

___________________________________________________________________________

**Table S2.** Mini pulse-amplitude modulator (MINI-PAM), Heinz Walz GmbH Germany, settings as used for all F_V_/F_M_ and rapid light curve (RLCs) measurements.

| **PAM setting** | **Value** |
| --- | --- |
| Measurement intensity (MI) | 8 |
| Saturation intensity (SI) | 8 |
| Saturation width (SW) | 0.8 |
| Actinic intensity (AI) | 5 |
| Actinic width (AW) | 0:30 |
| Actinic light factor (AF) | 1 |
| Gain (G) | 8 |
| Damp (D) | 2 |
| ETR factor (EF) | 0:84 |
| *F_o_* | 0 |
| Clock width (CW) | 0:30 |
| Clock item (CI) | 1 |
| Light curve width (LW) | 0:10 |
| Light curve intensity (LI) | 3 |
| Induction curve delay (ID) | 0:40 |
| Induction curve width (IW) | 0:20 |
| Temp offs (DO) | 0:00 |
| Temp gain (DG) | 1.00 |
| Light offs (LO) | 0:00 |
| Light Gain (LG) | 1.00 |
| Auto-Zero (FO | *60 |

*Auto-zero value was determined using the auto-zero setting along the side of the aquaria bucket in which the coral samples were kept, a non-photosynthetic surface, at the beginning of the sampling period.
