# Supplementary material for: Coral-dwelling fish moderate bleaching susceptibility of coral hosts
Source: PLoS One. 2018 Dec 14;13(12):e0208545. doi: 10.1371/journal.pone.0208545 (PMC6294555; doi:10.1371/journal.pone.0208545)
Supplement: S3 Text — (DOCX) [file pone.0208545.s003.docx]

**S3 Text:** Coral tissue analysis

*The following supplement accompanies the article*

Coral-dwelling fish moderate bleaching susceptibility of coral hosts

**List of authors**

TJ Chase^1,2^*, MS Pratchett^2^, GE Frank^1^, and MO Hoogenboom^1, 2^

___________________________________________________________________________

**S3 Text:** Coral tissue analysis

One coral fragment per colony, approximately 6cm in length was collected from each experimental colony at the end of the acclimation, stress, and recovery phases. Fragments (n=114 in total) were subsequently frozen with liquid nitrogen and transported to James Cook University for analysis. Tissue was removed from the skeleton using compressed air and 0.45μm filtered seawater, collected, and homogenized. The resulting tissue ‘slurries” were divided into aliquots for protein assays (1mL), symbiont counts (0.9ml of ‘slurry’ and 0.1ml of formaldehyde, to preserve samples), total chlorophyll (5ml), and tissue biomass (8mL). Coral skeletons were retained to quantify fragment surface area using a wax coating technique (1,2) as:

*Surface area (cm^2^) = Z x W*  (1)

Where Z is the regression equation for the standard calibration curve (cm^2^ x mg^-1^) and W is the difference in weight between wax coating (mg). *Symbiodinium* density was determined by 6 replicate counts of each homogenized (IKA T10 basic, Ultra Turrax Homogenizer) sample (1ml) using an improved Neubauer Haemocytometer (for 1 minute, Hirschmann EM, 0.100mm). Symbiodinium (zooxanthellae) density was calculated as follows:

*# symbiodinium cc^-3^ = N x 16^4^ x dilution factor* (2)

with N as the mean number of zooxanthellae counted in 25-medium-squares of the Hemocytometer. Chlorophyll was extracted by adding 4mL acetone to each sample and vortexing it for 30s to mix. Total chlorophyll (chl *a* + chl *c*, μg/ml^-1^) content was measured using spectrophotometry on a SpectraMex Plus^384^ Microplate reader (Molecular Devices). Total chlorophyll (chl *a* + chl *c*, μg/ml) was calculated (3) as:

*Chl a (ug/ml) = 11.43 (A_663_ – A_750_) – 0.64 (A_630_ – A_750_) +*

*Chl c (ug/ml) = -3.63 (A_663_ – A_750_) + 27.09 (A_630_ – A_750_)*  (3)

where A_630_, A_663_, and A_750_ are the absorbance at 630, 663, and 750nm. As coral fragments were not the sample size, measurements of *Symbiodinum* density and total chlorophyll were normalized by surface area and are reported per cm^2^. Total protein content (mg cm^-2^) was extracted using spectrophotometry from fragments into a buffered solution and quantified using the Red 660 protein assay and using bovine serum albumen protein (BAS) as a standard curve (4). The tissue biomass of the coral tissue was determined using the ash-free dry weight (AFDW) method by placing 8ml of the coral tissue ‘slurry’ into a freeze dryer (Christ, Alpaa 1-1 LO plus)) for 48 hours and then incinerated in a muffle furnace (Yokogawa model UP150 muffle furnace) at 550°C. The AFDW was calculated by subtracting the ash-weigh (AW) from dry weight (DF) and normalized per fragment surface area (5). All samples were allowed sufficient time to cool (~7 days) prior to measuring AW.

**References:**

1. Stimson J, Kinzie RA (1991) The temporal pattern and rate of release of zooxanthellae from the reef coral *Pocillopora damicornis* (Linnaeus) under nitrogen-enrichment and control conditions. Journal of Experimental Marine Biology 15:63-74
2. Vytopil E, Willis BL (2001) Epifaunal community structure in *Acropora* spp. (Scleractinian) on the Great Barrier Reef: implications of coral morphology and habitat complexity. Coral Reefs 20:281-288
3. Jeffrey SW, Humphrey GF (1975) New spectrophotometric equations for determining chlorophylls *a*, *b*, *c*_1_, and *c*_2_ in higher plants, algae and natural phytoplankton. Biochemie und Physiologie de Pflanzen 167:191-194
4. Palmer CV, Modi CK, Mydlarz LD (2009) Coral fluorescent proteins as antioxidants. PLoS ONE 4: e7298. Doi:10.1371/journal.pone.0007298
5. Leuven R, Brock TCM, van Druten HAM (1985) Effects of preservation on dry and ash-free dry-weight biomass of some aquatic macro-invertebrates. Hydrobiologia 127: 151-159
