# Supplementary material for: Coral-dwelling fish moderate bleaching susceptibility of coral hosts
Source: PLoS One. 2018 Dec 14;13(12):e0208545. doi: 10.1371/journal.pone.0208545 (PMC6294555; doi:10.1371/journal.pone.0208545)
Supplement: S1 Table — (PDF) [file pone.0208545.s004.pdf]

S1 Table. Raw data: mean photosynthetic yield (FV/FM) for Pocillopora damicornis colonies in aquaria bleaching experiment at Lizard Island Research Station during Acclimation and Stress time periods.

temperature	fish	time	mean	sd	count	se
ambient	fish	1	0.703	0.01194987	9	0.00398329
ambient	fish	3	0.689	0.01483052	9	0.00494351
ambient	fish	5	0.698	0.02414412	9	0.00804804
ambient	fish	9	0.694	0.01752811	9	0.0058427
ambient	fish	11	0.712	0.03318299	9	0.011061
ambient	fish	14	0.68	0.00507171	9	0.00169057
ambient	fish	16	0.68	0.00507171	9	0.00169057
ambient	fish	19	0.696	0.02026217	9	0.00675406
ambient	fish	22	0.696	0.02026217	9	0.00675406
ambient	fish	25	0.681	0.0246182	9	0.00820607
ambient	fish	29	0.69	0.0184344	9	0.0061448
ambient	fish	30	0.692	0.02263935	9	0.00754645
ambient	fish	31	0.691	0.01788932	9	0.00596311
ambient	fish	32	0.714	0.02445448	9	0.00815149
ambient	fish	33	0.713	0.03201697	9	0.01067232
ambient	fish	34	0.713	0.03201697	9	0.01067232
ambient	fish	35	0.708	0.02634816	9	0.00878272
ambient	fish	37	0.691	0.02825725	9	0.00941908
ambient	fish	38	0.691	0.02825725	9	0.00941908
ambient	nofish	1	0.705	0.01220137	9	0.00406712
ambient	nofish	3	0.686	0.0128648	9	0.00428827
ambient	nofish	5	0.687	0.00890242	9	0.00296747
ambient	nofish	9	0.671	0.04022303	9	0.01340768
ambient	nofish	11	0.686	0.02760384	9	0.00920128
ambient	nofish	14	0.67	0.02862357	9	0.00954119
ambient	nofish	16	0.67	0.02862357	9	0.00954119
ambient	nofish	19	0.692	0.01108817	9	0.00369606
ambient	nofish	22	0.692	0.01108817	9	0.00369606
ambient	nofish	25	0.666	0.02910427	9	0.00970142
ambient	nofish	29	0.669	0.01830967	9	0.00610322
ambient	nofish	30	0.686	0.02314754	9	0.00771585
ambient	nofish	31	0.681	0.02722018	9	0.00907339
ambient	nofish	32	0.691	0.02527186	9	0.00842395
ambient	nofish	33	0.697	0.02616867	9	0.00872289
ambient	nofish	34	0.697	0.02616867	9	0.00872289
ambient	nofish	35	0.69	0.02830587	9	0.00943529
ambient	nofish	37	0.676	0.03766474	9	0.01255491
ambient	nofish	38	0.676	0.03766474	9	0.01255491
hot	fish	1	0.692	0.01896708	10	0.00599792
hot	fish	3	0.685	0.01899082	10	0.00600542
hot	fish	5	0.689	0.02584355	10	0.00817245
hot	fish	9	0.668	0.03031929	10	0.0095878
hot	fish	11	0.685	0.02210304	10	0.0069896
hot	fish	14	0.664	0.01991305	10	0.00629706

hot	fish	16	0.676	0.01908749	10	0.00603599
hot	fish	19	0.654	0.01927386	10	0.00609493
hot	fish	22	0.677	0.02659472	10	0.00840999
hot	fish	25	0.641	0.02524634	10	0.00798359
hot	fish	29	0.621	0.08072028	10	0.025526
hot	fish	30	0.579	0.08673897	10	0.02742927
hot	fish	31	0.571	0.10375741	10	0.03281097
hot	fish	32	0.559	0.11346602	10	0.03588111
hot	fish	33	0.535	0.09772521	10	0.03090343
hot	fish	34	0.57	0.10342085	10	0.03270454
hot	fish	35	0.595	0.09391791	10	0.02969945
hot	fish	37	0.56	0.09813644	10	0.03103347
hot	fish	38	0.526	0.10220251	10	0.03231927
hot	nofish	1	0.704	0.02255391	9	0.00751797
hot	nofish	3	0.683	0.01019002	9	0.00339667
hot	nofish	5	0.676	0.01503278	9	0.00501093
hot	nofish	9	0.649	0.06629123	9	0.02209708
hot	nofish	11	0.664	0.04797974	9	0.01599325
hot	nofish	14	0.641	0.04595711	9	0.01531904
hot	nofish	16	0.661	0.01339672	9	0.00446558
hot	nofish	19	0.653	0.02159997	9	0.00719999
hot	nofish	22	0.634	0.03997986	9	0.01332662
hot	nofish	25	0.632	0.04865148	9	0.01621716
hot	nofish	29	0.591	0.056284	9	0.01876133
hot	nofish	30	0.539	0.06373774	9	0.02124592
hot	nofish	31	0.519	0.08539149	9	0.02846383
hot	nofish	32	0.483	0.06776323	9	0.02258774
hot	nofish	33	0.441	0.08376391	9	0.0279213
hot	nofish	34	0.432	0.10548644	9	0.03516215
hot	nofish	35	0.385	0.14773272	9	0.04924424
hot	nofish	37	0.344	0.11941303	9	0.03980434
hot	nofish	38	0.355	0.15134641	9	0.0504488
