# Supplementary material for: Coral-dwelling fish moderate bleaching susceptibility of coral hosts
Source: PLoS One. 2018 Dec 14;13(12):e0208545. doi: 10.1371/journal.pone.0208545 (PMC6294555; doi:10.1371/journal.pone.0208545)
Supplement: S2 Table — (PDF) [file pone.0208545.s005.pdf]

S2 Table. Raw data: mean photosynthetic yield (FV/FM) for Pocillopora damicornis colonies in aquaria bleaching experiment at Lizard Island Research Station during the Recovery period.

treatment	temperature	fish	time	mean	SE
RHN	hot	nofish	44	0.317	0.04890145
RHN	hot	nofish	47	0.33522222	0.05532923
RHN	hot	nofish	50	0.32485185	0.05190034
RHN	hot	nofish	54	0.29588889	0.04961699
RHN	hot	nofish	58	0.15833333	0.075
RHN	hot	nofish	62	0.1247037	0.0709
RHN	hot	nofish	64	0.11662963	0.06466658
RHF	hot	fish	44	0.5481	0.04912368
RHF	hot	fish	47	0.5652963	0.04926956
RHF	hot	fish	50	0.5741	0.04705374
RHF	hot	fish	54	0.53388889	0.06190229
RHF	hot	fish	58	0.5525	0.05570871
RHF	hot	fish	62	0.53106667	0.07277306
RHF	hot	fish	64	0.53936364	0.07506602
RAN	ambient	nofish	44	0.67858333	0.01119519
RAN	ambient	nofish	47	0.68159259	0.00754018
RAN	ambient	nofish	50	0.68644444	0.00638937
RAN	ambient	nofish	54	0.67188889	0.00664301
RAN	ambient	nofish	58	0.69403704	0.00631319
RAN	ambient	nofish	62	0.67959259	0.00688638
RAN	ambient	nofish	64	0.67744444	0.01006706
RAF	ambient	fish	44	0.6852963	0.01180166
RAF	ambient	fish	47	0.71225926	0.01262759
RAF	ambient	fish	50	0.70307407	0.00703207
RAF	ambient	fish	54	0.70462963	0.00402709
RAF	ambient	fish	58	0.72785185	0.00704069
RAF	ambient	fish	62	0.72577778	0.00710851
RAF	ambient	fish	64	0.72051852	0.00861561
