# Supplementary material for: Coral-dwelling fish moderate bleaching susceptibility of coral hosts
Source: PLoS One. 2018 Dec 14;13(12):e0208545. doi: 10.1371/journal.pone.0208545 (PMC6294555; doi:10.1371/journal.pone.0208545)
Supplement: S3 Table — (PDF) [file pone.0208545.s006.pdf]

S3 Table. Raw data: coral tissue compositions for Pocillopora damicornis colonies in aquaria bleaching experiment at Lizard Island Research Station.

experiment	phase	temperature	treatment	sample	zooxs	chl	protein	ash
bleaching in tar	acclimation	ambient	nofish	1	1.816607597	1.111879846	0.798212821	0.001283998
bleaching in tar	acclimation	ambient	fish	2	0.989205655	2.044137404	0.449864199	0.000572166
bleaching in tar	acclimation	ambient	nofish	3	0.639242273	1.583990623	0.332252568	0.000893485
bleaching in tar	acclimation	ambient	fish	4	1.060881254	1.986921938	0.718512086	0.000579025
bleaching in tar	acclimation	ambient	nofish	5	0.792844387	0.338666124	0.529837404	0.001175088
bleaching in tar	acclimation	ambient	nofish	6	0.624480675	2.3605538	0.611552716	0.000597589
bleaching in tar	acclimation	ambient	fish	7	1.897208445	3.067528349	1.016079316	0.000842306
bleaching in tar	acclimation	ambient	fish	8	1.277705621	2.301775232	0.871579748	0.000849479
bleaching in tar	acclimation	ambient	nofish	9	1.112300363	1.576898858	0.684073008	0.000737959
bleaching in tar	acclimation	ambient	fish	10	0.860033785	1.229830507	0.604127973	0.000384486
bleaching in tar	acclimation	hot	fish	13	1.024048126	1.239890617	0.468406554	0.000700109
bleaching in tar	acclimation	hot	fish	14	1.109743007	3.231695089	0.532864827	0.000862817
bleaching in tar	acclimation	hot	nofish	15	0.843434343	1.450347847	0.655577153	0.000417341
bleaching in tar	acclimation	hot	nofish	16	1.062362966	1.337356242	0.997415175	0.002242829
bleaching in tar	acclimation	hot	fish	17	0.380460357	1.066623855	0.302487721	0.000806926
bleaching in tar	acclimation	hot	nofish	18	0.787364001	1.219285264	0.441809154	0.000722027
bleaching in tar	acclimation	hot	fish	19	1.243603758	1.037244077	0.442666448	0.000631339
bleaching in tar	acclimation	hot	nofish	20	0.811809033	1.154246319	0.687012556	0.00071055
bleaching in tar	acclimation	hot	nofish	21	0.707455401	1.082678957	0.366817952	0.000474137
bleaching in tar	acclimation	hot	fish	22	1.084204847	1.242918505	0.534969497	0.000680808
bleaching in tar	acclimation	hot	nofish	23	0.796323677	1.078130853	0.547367448	0.000486816
bleaching in tar	acclimation	hot	fish	24	0.780058715	1.391333816	0.583169753	0.000455883
bleaching in tar	acclimation	hot	fish	25	0.784736868	1.475769407	0.696970245	0.000837584
bleaching in tar	acclimation	hot	nofish	26	0.907450374	1.480693852	0.684912688	0.000594988
bleaching in tar	acclimation	hot	nofish	27	0.826958398	1.305839251	0.676266376	0.000892273
bleaching in tar	acclimation	hot	fish	28	1.053939846	0.688701146	0.673685205	0.000943893
bleaching in tar	acclimation	hot	fish	29	0.712358283	2.783113744	0.676138478	0.000990092
bleaching in tar	acclimation	hot	nofish	30	1.798068361	2.374029838	1.60702043	0.001614298
bleaching in tar	acclimation	ambient	fish	31	1.124034138	2.135194451	0.503575517	0.000755391
bleaching in tar	acclimation	ambient	nofish	32	0.896448127	1.439366907	0.667627219	0.000833929
bleaching in tar	acclimation	ambient	nofish	34	0.532617278	1.244319924	0.559471872	0.000376378
bleaching in tar	acclimation	ambient	fish	35	1.012664308	1.84989363	0.690331005	0.000636321
bleaching in tar	acclimation	ambient	nofish	36	1.370472986	1.390490675	0.669427223	0.000620275
bleaching in tar	acclimation	ambient	fish	38	0.927643785	1.341770515	0.463903359	0.000689904
bleaching in tar	acclimation	ambient	nofish	39	1.023289758	1.772199664	0.834746788	0.000889548
bleaching in tar	acclimation	ambient	fish	40	1.009848105	0.882080183	0.518649624	0.00062321
bleaching in tar	acclimation	hot	fish	41	0.996789381	1.454637565	0.603825254	0.000494453
bleaching in tar	recovery	ambient	nofish	1	1.992642551	5.151888547	0.121573229	0.001379035
bleaching in tar	recovery	ambient	fish	2	3.453724289	4.813345254	0.133379578	0.001752695
bleaching in tar	recovery	ambient	nofish	3	2.008694475	2.291532972	0.064650696	0.000656526
bleaching in tar	recovery	ambient	fish	4	2.431941924	5.159107078	0.128003269	0.00089265
bleaching in tar	recovery	ambient	nofish	5	1.950947603	3.029310357	0.091087289	0.000960013
bleaching in tar	recovery	ambient	nofish	6	1.149986969	2.873549941	0.082393115	0.001485106
bleaching in tar	recovery	ambient	fish	7	2.684011399	4.227083579	0.085539212	0.001114829
bleaching in tar	recovery	ambient	fish	8	2.600378788	4.910916733	0.119431066	0.00079742
bleaching in tar	recovery	ambient	nofish	9	1.534489799	1.86138845	0.070132155	0.001389457
bleaching in tar	recovery	ambient	fish	10	2.691625375	2.805215269	0.108592743	0.001103982
bleaching in tar	recovery	hot	fish	13	0.215203624	0.225883103	0.03277936	0.000501086
bleaching in tar	recovery	hot	fish	14	1.032298513	2.15680736	0.057393664	0.000632837
bleaching in tar	recovery	hot	nofish	15	0.029294586	0.038510224	0.021114089	0.000718538
bleaching in tar	recovery	hot	nofish	16	0.507976744	0.620874298	0.041334938	0.000501901
bleaching in tar	recovery	hot	fish	17	0.026154458	0.629608782	0.047026061	0.001507532
bleaching in tar	recovery	hot	nofish	18	0.063345418	0.147944868	0.018829369	0.00067821
bleaching in tar	recovery	hot	fish	19	1.388412753	3.571317707	0.110677985	0.000839835

bleaching in tar recovery	hot	nofish	20	0.026406591	0.041652172	0.026056835	0.00055644
bleaching in tar recovery	hot	nofish	21	0	0.564902449	0.033393659	0.001118935
bleaching in tar recovery	hot	fish	22	0.212279171	0.199685974	0.036805209	0.000648619
bleaching in tar recovery	hot	nofish	23	0.307947412	0.49725	0.03796397	0.000574452
bleaching in tar recovery	hot	fish	24	0.808983467	1.378572881	0.050239928	0.000736282
bleaching in tar recovery	hot	fish	25	2.021007168	4.879486212	0.090020131	0.0010077
bleaching in tar recovery	hot	nofish	26	0	0.105235533	0.027985049	0.00128996
bleaching in tar recovery	hot	nofish	27	0	0.109245963	0.018781895	0.000688188
bleaching in tar recovery	hot	fish	28	0.035107429	0.134088436	0.241345806	0.000649501
bleaching in tar recovery	hot	fish	29	0	0.189857997	0.249745095	0.000807847
bleaching in tar recovery	hot	nofish	30	0	0.264608659	0.270508426	0.001056666
bleaching in tar recovery	ambient	fish	31	2.890185069	3.730214162	1.45092678	0.000927925
bleaching in tar recovery	ambient	nofish	32	0.723742277	1.302519241	1.073785693	0.000923989
bleaching in tar recovery	ambient	nofish	34	1.515660492	2.071501944	1.427048762	0.001910308
bleaching in tar recovery	ambient	fish	35	1.87677378	4.982120897	1.370816733	0.00100079
bleaching in tar recovery	ambient	nofish	36	1.023323591	2.411985135	0.757673759	0.000602439
bleaching in tar recovery	ambient	fish	38	1.226227595	1.906582381	0.891392515	0.001341803
bleaching in tar recovery	ambient	nofish	39	1.047670509	1.633104872	0.898388734	0.000961924
bleaching in tar recovery	ambient	fish	40	1.101089892	2.255079884	1.08760643	0.000929995
bleaching in tar recovery	hot	fish	41	0.296529755	0.251337021	0.690730819	0.00074573
bleaching in tar stress	ambient	nofish	1	0.726021289	1.179447952	0.473686486	0.000428067
bleaching in tar stress	ambient	fish	2	1.28106977	2.20924265	0.76715481	0.000463712
bleaching in tar stress	ambient	nofish	3	1.022549676	2.046800427	0.364389097	0.000725546
bleaching in tar stress	ambient	fish	4	1.152740814	1.186269582	0.664840985	0.000443481
bleaching in tar stress	ambient	nofish	5	0.271675997	2.324595613	0.459848346	0.001003564
bleaching in tar stress	ambient	nofish	6	0.672252871	2.391492517	0.564589912	0.000153036
bleaching in tar stress	ambient	fish	7	1.202337226	1.761391711	0.582659281	0.00058008
bleaching in tar stress	ambient	fish	8	1.381736685	0.126462404	0.862014159	0.000507697
bleaching in tar stress	ambient	nofish	9	1.097378088	1.255340704	0.826394532	0.000724636
bleaching in tar stress	ambient	fish	10	1.061389304	0.010454713	0.403359333	0.000579666
bleaching in tar stress	hot	fish	13	0.114307605	0.496395999	0.367866816	0.000460026
bleaching in tar stress	hot	fish	14	0.738383821	1.431827964	0.574066617	0.000411992
bleaching in tar stress	hot	nofish	15	0.054546446	0.551203379	0.287042256	0.000478645
bleaching in tar stress	hot	nofish	16	0.345873464	0.008581203	0.579922901	0.000375379
bleaching in tar stress	hot	fish	17	0.391544583	0.393766947	0.554545544	0.000579802
bleaching in tar stress	hot	nofish	18	0.029791511	1.258680303	0.306390421	0.000410999
bleaching in tar stress	hot	fish	19	0.672939899	0.435713096	0.636008142	0.000556176
bleaching in tar stress	hot	nofish	20	0.060054836	0.033681506	0.350000302	0.000502659
bleaching in tar stress	hot	nofish	21	0.121243865	0.093919353	0.209357719	0.000381045
bleaching in tar stress	hot	fish	22	0.4308049	0.886104442	0.441200305	0.000317018
bleaching in tar stress	hot	nofish	23	0.245446685	0.375495461	0.447127554	0.0002449
bleaching in tar stress	hot	fish	24	0.471159169	1.686999545	0.059985542	0.000370826
bleaching in tar stress	hot	fish	25	0.686554215	0.855793292	0.612062419	0.000227894
bleaching in tar stress	hot	nofish	26	0.253256774	1.505113056	0.434135463	0.000331247
bleaching in tar stress	hot	nofish	27	0.007164248	0.265471252	0.284800952	0.000276289
bleaching in tar stress	hot	fish	28	0.757930115	0.007883431	0.479062351	0.000384462
bleaching in tar stress	hot	fish	29	0.162260026	1.148869634	0.350329217	0.000741743
bleaching in tar stress	hot	nofish	30	0.060621239	0.047583285	0.363108256	0.000239613
bleaching in tar stress	ambient	fish	31	1.195746444	1.529970948	0.989548501	0.000558852
bleaching in tar stress	ambient	nofish	32	1.367129053	2.318361563	1.092932225	0.000471164
bleaching in tar stress	ambient	nofish	34	1.1122391	2.612440571	0.442842349	0.000212629
bleaching in tar stress	ambient	fish	35	1.972780274	3.309792069	1.0540203	0.000587044
bleaching in tar stress	ambient	nofish	36	0.889555127	1.628754535	0.440457423	0.000532177
bleaching in tar stress	ambient	fish	38	0.942450491	2.303928848	0.435357461	0.000377073
bleaching in tar stress	ambient	nofish	39	0.792708307	0.456637676	0.536690425	0.000745034

bleaching in tar stress	ambient	fish	40	0.241512157	0.48443233	0.550023873	7.52E-05
bleaching in tar stress	hot	fish	41	0.214696309	0.203492936	0.257718324	0.000224704
