# Supplementary material for: Coral-dwelling fish moderate bleaching susceptibility of coral hosts
Source: PLoS One. 2018 Dec 14;13(12):e0208545. doi: 10.1371/journal.pone.0208545 (PMC6294555; doi:10.1371/journal.pone.0208545)
Supplement: S4 Table — (PDF) [file pone.0208545.s007.pdf]

S4 Table. Raw data: coral tissue compositions for *in situ Pocillopora damicornis* colonies around Lizard Island, under non-bleaching conditions.

coral	treatment	fishno	chl	zoox	protein	ash
FF1	fish	10	1.24376874	1.41712184	0.84402109	0.00069024
FF2	fish	6	1.84382858	1.43343134	1.5539055	0.0006895
FF3	fish	4	0.9126946	2.09921698	0.66741295	0.00131149
FF4	fish	4	1.57105363	0.80669675	0.90782991	0.00075966
FF5	fish	3	1.04821269	1.62875321	0.84713475	0.00036301
NFF1	nofish	0	0.84668299	1.25400992	0.78751186	0.00056558
NFF2	nofish	0	0.9638373	0.78653783	0.5917362	0.00068764
NNF3	nofish	0	0.48488897	0.61945605	0.78319254	0.00101836
NFF4	nofish	0	0.22277997	0.72291934	0.74556688	0.00080636
NFF5	nofish	0	1.07577057	0.30830979	0.3770942	0.0005596
