# Supplementary material for: Coral-dwelling fish moderate bleaching susceptibility of coral hosts
Source: PLoS One. 2018 Dec 14;13(12):e0208545. doi: 10.1371/journal.pone.0208545 (PMC6294555; doi:10.1371/journal.pone.0208545)
Supplement: S5 Table — (PDF) [file pone.0208545.s008.pdf]

S5 Table. Raw data: coral tissue compositions for in situ Seriatopora hystrix colonies around Lizard Island, during a bleaching event.

| experiment | phase | site | treatment | colony | daruanus | tissue |
|---|---|---|---|---|---|---|
| shystrix_field | field | mangrove | fish | 1 | 5 | symbiodinidu |
| shystrix_field | field | mangrove | nofish | 2 | 0 | symbiodinidu |
| shystrix_field | field | mangrove | fish | 3 | 2 | symbiodinidu |
| shystrix_field | field | mangrove | nofish | 5 | 0 | symbiodinidu |
| shystrix_field | field | mangrove | fish | 6 | 3 | symbiodinidu |
| shystrix_field | field | mangrove | nofish | 7 | 0 | symbiodinidu |
| shystrix_field | field | mangrove | fish | 8 | 6 | symbiodinidu |
| shystrix_field | field | mangrove | fish | 9 | 4 | symbiodinidu |
| shystrix_field | field | mangrove | nofish | 10 | 0 | symbiodinidu |
| shystrix_field | field | loomis | fish | 11 | 3 | symbiodinidu |
| shystrix_field | field | loomis | nofish | 12 | 0 | symbiodinidu |
| shystrix_field | field | loomis | nofish | 13 | 0 | symbiodinidu |
| shystrix_field | field | loomis | fish | 14 | 4 | symbiodinidu |
| shystrix_field | field | loomis | fish | 15 | 5 | symbiodinidu |
| shystrix_field | field | loomis | nofish | 16 | 0 | symbiodinidu |
| shystrix_field | field | loomis | fish | 17 | 3 | symbiodinidu |
| shystrix_field | field | loomis | fish | 18 | 3 | symbiodinidu |
| shystrix_field | field | loomis | nofish | 19 | 0 | symbiodinidu |
| shystrix_field | field | loomis | nofish | 20 | 0 | symbiodinidu |
| shystrix_field | field | palfreypatch | fish | 21 | 2 | symbiodinidu |
| shystrix_field | field | palfreypatch | fish | 22 | 5 | symbiodinidu |
| shystrix_field | field | palfreypatch | nofish | 23 | 0 | symbiodinidu |
| shystrix_field | field | palfreypatch | nofish | 24 | 0 | symbiodinidu |
| shystrix_field | field | palfreypatch | fish | 25 | 2 | symbiodinidu |
| shystrix_field | field | palfreypatch | fish | 27 | 8 | symbiodinidu |
| shystrix_field | field | palfreypatch | fish | 28 | 2 | symbiodinidu |
| shystrix_field | field | palfreypatch | nofish | 29 | 0 | symbiodinidu |
| shystrix_field | field | palfreypatch | nofish | 30 | 0 | symbiodinidu |
| shystrix_field | field | picnicbeach | fish | 31 | 3 | symbiodinidu |
| shystrix_field | field | picnicbeach | fish | 32 | 1 | symbiodinidu |
| shystrix_field | field | picnicbeach | nofish | 33 | 0 | symbiodinidu |
| shystrix_field | field | picnicbeach | fish | 34 | 2 | symbiodinidu |
| shystrix_field | field | picnicbeach | nofish | 35 | 0 | symbiodinidu |
| shystrix_field | field | picnicbeach | fish | 36 | 2 | symbiodinidu |
| shystrix_field | field | picnicbeach | nofish | 37 | 0 | symbiodinidu |
| shystrix_field | field | picnicbeach | fish | 38 | 4 | symbiodinidu |
| shystrix_field | field | picnicbeach | nofish | 39 | 0 | symbiodinidu |
| shystrix_field | field | picnicbeach | nofish | 40 | 0 | symbiodinidu |
| shystrix_field | field | mangrove | fish | 1 | 5 | chlorophyll |
| shystrix_field | field | mangrove | fish | 3 | 2 | chlorophyll |
| shystrix_field | field | mangrove | nofish | 5 | 0 | chlorophyll |
| shystrix_field | field | mangrove | fish | 6 | 3 | chlorophyll |
| shystrix_field | field | mangrove | nofish | 7 | 0 | chlorophyll |
| shystrix_field | field | mangrove | fish | 8 | 6 | chlorophyll |

| | | | | | | |
|---|---|---|---|---|---|---|
| shystrix_field | field | mangrove | fish | 9 | 4 | chlorophyll |
| shystrix_field | field | mangrove | nofish | 10 | 0 | chlorophyll |
| shystrix_field | field | loomis | fish | 11 | 3 | chlorophyll |
| shystrix_field | field | loomis | nofish | 12 | 0 | chlorophyll |
| shystrix_field | field | loomis | nofish | 13 | 0 | chlorophyll |
| shystrix_field | field | loomis | fish | 14 | 4 | chlorophyll |
| shystrix_field | field | loomis | fish | 15 | 5 | chlorophyll |
| shystrix_field | field | loomis | nofish | 16 | 0 | chlorophyll |
| shystrix_field | field | loomis | fish | 17 | 3 | chlorophyll |
| shystrix_field | field | loomis | fish | 18 | 3 | chlorophyll |
| shystrix_field | field | loomis | nofish | 19 | 0 | chlorophyll |
| shystrix_field | field | loomis | nofish | 20 | 0 | chlorophyll |
| shystrix_field | field | palfrey patch | fish | 21 | 2 | chlorophyll |
| shystrix_field | field | palfrey patch | fish | 22 | 5 | chlorophyll |
| shystrix_field | field | palfrey patch | nofish | 23 | 0 | chlorophyll |
| shystrix_field | field | palfrey patch | nofish | 24 | 0 | chlorophyll |
| shystrix_field | field | palfrey patch | fish | 25 | 2 | chlorophyll |
| shystrix_field | field | palfrey patch | fish | 27 | 8 | chlorophyll |
| shystrix_field | field | palfrey patch | fish | 28 | 2 | chlorophyll |
| shystrix_field | field | palfrey patch | nofish | 29 | 0 | chlorophyll |
| shystrix_field | field | palfrey patch | nofish | 30 | 0 | chlorophyll |
| shystrix_field | field | picnic beach | fish | 31 | 3 | chlorophyll |
| shystrix_field | field | picnic beach | nofish | 32 | 1 | chlorophyll |
| shystrix_field | field | picnic beach | nofish | 33 | 0 | chlorophyll |
| shystrix_field | field | picnic beach | fish | 34 | 2 | chlorophyll |
| shystrix_field | field | picnic beach | nofish | 35 | 0 | chlorophyll |
| shystrix_field | field | picnic beach | fish | 36 | 2 | chlorophyll |
| shystrix_field | field | picnic beach | nofish | 37 | 0 | chlorophyll |
| shystrix_field | field | picnic beach | fish | 38 | 4 | chlorophyll |
| shystrix_field | field | picnic beach | nofish | 39 | 0 | chlorophyll |
| shystrix_field | field | picnic beach | nofish | 40 | 0 | chlorophyll |
| shystrix_field | field | mangrove | fish | 1 | 5 | protein |
| shystrix_field | field | mangrove | nofish | 2 | 0 | protein |
| shystrix_field | field | mangrove | fish | 3 | 2 | protein |
| shystrix_field | field | mangrove | nofish | 5 | 0 | protein |
| shystrix_field | field | mangrove | fish | 6 | 3 | protein |
| shystrix_field | field | mangrove | nofish | 7 | 0 | protein |
| shystrix_field | field | mangrove | fish | 8 | 6 | protein |
| shystrix_field | field | mangrove | fish | 9 | 4 | protein |
| shystrix_field | field | mangrove | nofish | 10 | 0 | protein |
| shystrix_field | field | loomis | fish | 11 | 3 | protein |
| shystrix_field | field | loomis | nofish | 12 | 0 | protein |
| shystrix_field | field | loomis | nofish | 13 | 0 | protein |
| shystrix_field | field | loomis | fish | 14 | 4 | protein |
| shystrix_field | field | loomis | fish | 15 | 5 | protein |

| | | | | | | |
|---|---|---|---|---|---|---|
| shystrix_field | field | loomis | nofish | 16 | 0 | protein |
| shystrix_field | field | loomis | fish | 17 | 3 | protein |
| shystrix_field | field | loomis | fish | 18 | 3 | protein |
| shystrix_field | field | loomis | nofish | 19 | 0 | protein |
| shystrix_field | field | loomis | nofish | 20 | 0 | protein |
| shystrix_field | field | palfreypatch | fish | 21 | 2 | protein |
| shystrix_field | field | palfreypatch | fish | 22 | 5 | protein |
| shystrix_field | field | palfreypatch | nofish | 23 | 0 | protein |
| shystrix_field | field | palfreypatch | nofish | 24 | 0 | protein |
| shystrix_field | field | palfreypatch | fish | 25 | 2 | protein |
| shystrix_field | field | palfreypatch | fish | 27 | 8 | protein |
| shystrix_field | field | palfreypatch | fish | 28 | 2 | protein |
| shystrix_field | field | palfreypatch | nofish | 29 | 0 | protein |
| shystrix_field | field | palfreypatch | nofish | 30 | 0 | protein |
| shystrix_field | field | picnicbeach | fish | 31 | 3 | protein |
| shystrix_field | field | picnicbeach | fish | 32 | 1 | protein |
| shystrix_field | field | picnicbeach | nofish | 33 | 0 | protein |
| shystrix_field | field | picnicbeach | fish | 34 | 2 | protein |
| shystrix_field | field | picnicbeach | nofish | 35 | 0 | protein |
| shystrix_field | field | picnicbeach | fish | 36 | 2 | protein |
| shystrix_field | field | picnicbeach | nofish | 37 | 0 | protein |
| shystrix_field | field | picnicbeach | fish | 38 | 4 | protein |
| shystrix_field | field | picnicbeach | nofish | 39 | 0 | protein |
| shystrix_field | field | picnicbeach | nofish | 40 | 0 | protein |

| concentration |
| --- |
| 0.33941071 |
| 0.08262869 |
| 0.33732869 |
| 0.24822232 |
| 0.37572146 |
| 0.22039945 |
| 0.19773168 |
| 0.30054487 |
| 0.1596285 |
| 0.47320613 |
| 0.25447329 |
| 0.39836519 |
| 0.41853346 |
| 0.38565431 |
| 0.18115942 |
| 0.36168762 |
| 0.75956908 |
| 0.56571124 |
| 0.2945319 |
| 0.41094327 |
| 0.37522679 |
| 0.21168698 |
| 0.25331162 |
| 0.39125656 |
| 0.36250647 |
| 0.28062799 |
| 0.23539781 |
| 0.18009565 |
| 0.29237677 |
| 0.30376355 |
| 0.24719101 |
| 0.3727491 |
| 0.4045811 |
| 0.03987197 |
| 0.40545204 |
| 0.38582656 |
| 0.29752109 |
| 0.19145974 |
| 0.21237649 |
| 0.28893977 |
| 0.18698199 |
| 0.22341438 |
| 0.12735612 |
| 0.15619031 |

| |
|---|
| 0.15706345 |
| 0.22787005 |
| 0.27197453 |
| 0.15923408 |
| 0.19327844 |
| 0.37924369 |
| 0.22560172 |
| 0.13888127 |
| 0.19049898 |
| 0.41125639 |
| 0.18072171 |
| 0.19545882 |
| 0.3787754 |
| 0.401875 |
| 0.14816809 |
| 0.20684968 |
| 0.19566696 |
| 0.26102958 |
| 0.16560227 |
| 0.21013028 |
| 0.2567271 |
| 0.17917476 |
| 0.15775493 |
| 0.10758034 |
| 0.2423 |
| 0.20861765 |
| 0.03720561 |
| 0.26844419 |
| 0.3267703 |
| 0.15095954 |
| 0.07427741 |
| 0.45669563 |
| 0.27719565 |
| 0.34346895 |
| 1.15943281 |
| 1.3351345 |
| 0.66126035 |
| 1.19340612 |
| 3.44163562 |
| 0.65239991 |
| 1.59116943 |
| 0.48441678 |
| 0.75965561 |
| 1.76913338 |
| 0.71580449 |

| |
|---|
| 0.3452683 |
| 0.68378036 |
| 0.04056357 |
| 0.27305035 |
| 0.91981408 |
| 0.80478084 |
| 1.97596676 |
| 0.31068223 |
| 0.15297131 |
| 1.05557577 |
| 0.96070352 |
| 1.55832136 |
| 0.55600322 |
| 0.67110159 |
| 0.53207006 |
| 0.74821434 |
| 0.47220916 |
| 0.62402199 |
| 0.57603509 |
| 2.38473392 |
| 0.41325834 |
| 1.59176238 |
| 3.0599495 |
| 0.63041158 |
