# Supplementary material for: Coral-dwelling fish moderate bleaching susceptibility of coral hosts
Source: PLoS One. 2018 Dec 14;13(12):e0208545. doi: 10.1371/journal.pone.0208545 (PMC6294555; doi:10.1371/journal.pone.0208545)
Supplement: S6 Table — Each of the 12 comparisons are completed for four coral tissue parameters: Symbiodinium, total chlorophyll, protein, and tissue biomass. For each comparison, the upper and lower confidence intervals and adjusted p-value is listed. (DOCX) [file pone.0208545.s009.docx]

**S6 Table:** Results of multiple selected comparisons (n=12) as a post hoc test for the linear mixed effects model of the effects of phase, temperature, and fish presence (*D. aruanus*) on *P. damicornis* colonies.

*The following supplement accompanies the article*

Coral-dwelling fish moderate bleaching susceptibility of coral hosts

**List of authors**

TJ Chase^1,2^*, MS Pratchett^2^, GE Frank^1^, and MO Hoogenboom^1, 2^

___________________________________________________________________________

**S6 Table.** Results of multiple selected comparisons (n=12) as a post hoc test for the linear mixed effects model of the effects of phase, temperature, and fish presence (*D. aruanus*) on *P. damicornis* colonies. Each of the 12 comparisons are completed for four coral tissue parameters: *Symbiodinium*, total chlorophyll, total proteins, and tissue biomass. For each comparison the upper and lower confidence intervals and adjusted p-value is listed.

| **Coral Tissue** | **Phase** | **Comparison** | **CI lower** | **CI upper** | **Adjusted p-value** |
| --- | --- | --- | --- | --- | --- |
| ***Symbiodinium*** | *Acclimation* | AAF vs. AHF | -0.17585 | 0.59946 | 0.699 |
|  |  | AAF vs. AAN | -0.24763 | 0.54783 | 0.877 |
|  |  | AHF vs AHN | -0.21795 | 0.57750 | 0.998 |
|  |  | AHF vs AAN | -0.44937 | 0.32595 | 0.989 |
|  |  | AAF vs AHN | -0.41969 | 0.35563 | 0.806 |
|  |  | AAN vs AHN | -0.36806 | 0.42740 | 0.999 |
|  | *Stress* | SHF vs. SHN | -0.05449 | 0.72083 | 0.265 |
|  |  | SAF vs SHF | 0.30737 | 1.08268 | **0.001** |
|  |  | SAF vs SAN | -0.12215 | 0.67331 | 0.450 |
|  |  | SAN vs SHN | 0.35488 | 1.15034 | **<0.001** |
|  | *Recovery* | RHF vs RHN | 0.1120506 | 0.88737 | **0.021** |
|  |  | RAF vs RAN | 0.49213 | 1.28759 | **<0.001** |
| **Total chlorophyll** | *Acclimation* | AAF vs. AHF | -0.48496 | 1.08269 | 0.874 |
|  |  | AAF vs. AAN | -0.34512 | 1.26297 | 0.669 |
|  |  | AHF vs AHN | -0.32092 | 1.28904 | 0.966 |
|  |  | AHF vs AAN | -0.62108 | 0.94118 | 0.977 |
|  |  | AAF vs AHN | -0.59863 | 0.96901 | 0.631 |
|  |  | AAN vs AHN | -0.77891 | 0.82918 | 1.000 |
|  | *Stress* | SHF vs. SHN | -0.11038 | 1.46360 | 0.262 |
|  |  | SAF vs SHF | 0.42775 | 2.00174 | **0.008** |
|  |  | SAF vs SAN | -0.17445 | 1.42768 | 0.342 |
|  |  | SAN vs SHN | 0.46366 | 2.06580 | **0.007** |
|  | *Recovery* | RHF vs RHN | 0.40809 | 1.97573 | **0.005** |
|  |  | RAF vs RAN | 0.55955 | 2.16765 | **<0.002** |
| **Total proteins** | *Acclimation* | AAF vs. AHF | -0.16955 | 0.363537 | 0.888 |
|  |  | AAF vs. AAN | -0.25686 | 0.290069 | 0.999 |
|  |  | AHF vs AHN | -0.36542 | 0.181513 | 0.945 |
|  |  | AHF vs AAN | -0.34694 | 0.186149 | 0.932 |
|  |  | AAF vs AHN | -0.45549 | 0.077594 | 0.909 |
|  |  | AAN vs AHN | -0.38202 | 0.164911 | 0.860 |
|  | *Stress* | SHF vs. SHN | -0.19569 | 0.337395 | 0.939 |
|  |  | SAF vs SHF | 0.00117 | 0.534255 | **0.046** |
|  |  | SAF vs SAN | -0.15045 | 0.396483 | 0.765 |
|  |  | SAN vs SHN | -0.05792 | 0.489016 | 0.337 |
|  | *Recovery* | RHF vs RHN | -0.00015 | 0.386359 | 0.596 |
|  |  | RAF vs RAN | -0.00019 | 0.361128 | 0.774 |
| **Tissue biomass** | *Acclimation* | AAF vs. AHF | -0.00036 | 0.00020 | 0.941 |
|  |  | AAF vs. AAN | -0.00046 | 0.00017 | 0.678 |
|  |  | AHF vs AHN | -0.00054 | 0.00001 | 0.652 |
|  |  | AHF vs AAN | -0.00037 | 0.00020 | 0.938 |
|  |  | AAF vs AHN | -0.00045 | 0.00012 | 0.331 |
|  |  | AAN vs AHN | -0.00037 | 0.00021 | 0.942 |
|  | *Stress* | SHF vs. SHN | -0.00022 | 0.00035 | 0.955 |
|  |  | SAF vs SHF | -0.00025 | 0.00032 | 0.992 |
|  |  | SAF vs SAN | -0.00038 | 0.00020 | 0.904 |
|  |  | SAN vs SHN | -0.00009 | 0.00049 | 0.478 |
|  | *Recovery* | RHF vs RHN | 0.00039 | 0.00015 | **0.012** |
|  |  | RAF vs RAN | -0.00034 | 0.00025 | 0.941 |
