# Supplementary material for: Coral-dwelling fish moderate bleaching susceptibility of coral hosts
Source: PLoS One. 2018 Dec 14;13(12):e0208545. doi: 10.1371/journal.pone.0208545 (PMC6294555; doi:10.1371/journal.pone.0208545)
Supplement: S7 Table — (DOCX) [file pone.0208545.s010.docx]

**S7 Table:** Comparison of linear (mx, b) and non-linear (mx, x0, w, a) regression equation and coefficients for photosynthetic efficiency (F_V_/F_M_) during Acclimation/Stress phase and Recovery phase for coral colonies under ambient and heated temperatures and with and without fish treatments.

*The following supplement accompanies the article*

Coral-dwelling fish moderate bleaching susceptibility of coral hosts

**List of authors**

TJ Chase^1,2^*, MS Pratchett^2^, GE Frank^1^, and MO Hoogenboom^1, 2^

___________________________________________________________________________

**S7 Table.** Comparison of linear (mx, b) and non-linear (mx, x0, w, a) regression equation and coefficients for photosynthetic efficiency (F_V_/F_M_) during Acclimation/Stress phase and Recovery phase for coral colonies under ambient and heated temperatures and with and without fish treatments.

| **Phase** | **Temperature** | **Fish Treatment** | **Equation** |
| --- | --- | --- | --- |
| Acclimation & Stress | Ambient | Fish | F_V_/F_M_ =0.0002x + 0.6929 \| R=0.0302 |
| Acclimation & Stress | Ambient | No fish | F_V_/F_M_ = -7E-5x + 0.6851 \| R=0.0053 |
| Acclimation & Stress | Hot | Fish | F_V_/F_M_ = (0.13019x + 0.54820) - (0.13019/1+exp(-(time-27.86174)/2.52201)) |
| Acclimation & Stress | Hot | No fish | F_V_/F_M_ = (0.35775x + 0.30696) - (0.35775/1+exp(-(time-31.91131)/2.73373)) |
| Recovery | Ambient | Fish | F_V_/F_M_ =0.0016x+0.6234 \| R=0.67068 |
| Recovery | Ambient | No fish | F_V_/F_M_ =9E-6x + 0.6809 \| R=9.8E-5 |
| Recovery | Hot | Fish | F_V_/F_M_ = -0.0013x + 0.6169 \| R=0.34805 |
| Recovery | Hot | No Fish | F_V_/F_M_ = -0.0123x + 0.9074 \| R=0.86893 |
