# Supplementary material for: Coral-dwelling fish moderate bleaching susceptibility of coral hosts
Source: PLoS One. 2018 Dec 14;13(12):e0208545. doi: 10.1371/journal.pone.0208545 (PMC6294555; doi:10.1371/journal.pone.0208545)
Supplement: S8 Table — Akaike’s information criteria (AIC) and AIC differences (ΔAIC) were calculated per model selection practice of Burnham and Anderson (2002) and Hoogenboom et al. (2011). Constructing the model with means (mean models presented in results), allows for regressions to explain a greater amount of variation in the data, compared with using all the individual points, but reduced statistical power. Data fitted through individual points yield similar results as mean models. (DOCX) [file pone.0208545.s011.docx]

**S8 Table:** Comparison of regression models testing the effects of temperature (ambient: 25°C or hot: 32°C) and fish presence (fish or no fish) on *P. damicornis* photosynthetic efficiency (F_V_/F_M_) through fitting the data points for each individual colony within treatments for F_V_/F_M_ associated with Acclimation and Stress experimental periods.

*The following supplement accompanies the article*

Coral-dwelling fish moderate bleaching susceptibility of coral hosts

**List of authors**

TJ Chase^1,2^*, MS Pratchett^2^, GE Frank^1^, and MO Hoogenboom^1, 2^

___________________________________________________________________________

**S8 Table.** Comparison of regression models testing the effects of temperature (ambient: 25°C or hot: 32°C) and fish presence (fish or no fish) on *P. damicornis* photosynthetic efficiency (F_V_/F_M_) through fitting the data points for each individual colony within treatments for F_V_/F_M_ associated with Acclimation and Stress experimental periods. Akaike’s information criteria (AIC) and AIC differences (ΔAIC) were calculated per model selection practice of Burnham and Anderson (2002) and Hoogenboom et al. (2011). Constructing the model with means (mean models presented in results), allows for regressions to explain a greater amount of variation in the data, compared with using all the individual points, but reduced statistical power. Data fitted through individual points yield similar results as mean models.

| **No.** | **Model** | **N** | **AIC** | **delta AIC** | **wAIC** |
| --- | --- | --- | --- | --- | --- |
| 1 | All data | 700 | -1378.48 | 1011.10 | 0.00 |
| 2 | By temperature treatment | 700 | -2258.90 | 130.67 | 0.00 |
| 3 | By fish treatment | 700 | -1466.06 | 923.52 | 0.00 |
| 4 | By temperature treatment by fish treatment | 700 | -2389.58 | 0.00 | 1.00 |
