# Supplementary material for: Coral-dwelling fish moderate bleaching susceptibility of coral hosts
Source: PLoS One. 2018 Dec 14;13(12):e0208545. doi: 10.1371/journal.pone.0208545 (PMC6294555; doi:10.1371/journal.pone.0208545)
Supplement: S1 Fig — (DOCX) [file pone.0208545.s012.docx]

**S1 Fig:** *In situ* *Seriatopora hystrix* sites in the Lizard Island lagoon.

*The following supplement accompanies the article*

Coral-dwelling fish moderate bleaching susceptibility of coral hosts

**List of authors**

TJ Chase^1,2^*, MS Pratchett^2^, GE Frank^1^, and MO Hoogenboom^1, 2^

___________________________________________________________________________

**S1 Fig.** Location of four *in situ* bleaching colonies (*S. hystrix*) within the Lizard Island Lagoon.
