# Supplementary material for: Coral-dwelling fish moderate bleaching susceptibility of coral hosts
Source: PLoS One. 2018 Dec 14;13(12):e0208545. doi: 10.1371/journal.pone.0208545 (PMC6294555; doi:10.1371/journal.pone.0208545)
Supplement: S2 Fig — FV/FM values were recorded post aquaria bleaching experiment, February 2016, when GBR: (a) FV/FM of coral colonies under new fish categories due to movement and additional fish species present, irrespective of past experimental treatments of heat and fish presence. New fish category includes aggregating fish (D. aruanus, D. reticulatus, P. amboinensis, and P. moluccensis) present during multiple observations. No fish SE = 0.0170, and Any fish SE = 0.0087. (b) FV/FM of coral colonies under category of only D. aruanus still present. D. aruanus absent SE = 0.0099, and D. aruanus present SE = 0.0126. (*) denotes a significant difference between fish treatments and error bars show SE. One-way analysis of variance (ANOVA) were performed on PAM data, 6-months post-experiment test for differences in FV/FM levels in field samples of P. damicornis. Data for FV/FM analysis met assumptions of normality (Shapiro-Wilks test) and homogeneity of variance (Bartlett’s test). (DOCX) [file pone.0208545.s013.docx]

**S2 Fig:** Photosynthetic yield for *Pocillopora damicornis* returned to the field.

*The following supplement accompanies the article*

Coral-dwelling fish moderate bleaching susceptibility of coral hosts

**List of authors**

TJ Chase^1,2^*, MS Pratchett^2^, GE Frank^1^, and MO Hoogenboom^1, 2^

___________________________________________________________________________

**S2 Fig** Differences in photosynthetic efficiency (F_V_/F_M_) of *P. damicornis* corals returned to the field, six months post aquaria bleaching experiment (February 2016, when GBR bleaching event was underway). **(a)** F_V_/F_M_ of coral colonies under new fish categories due to movement and additional fish species present, irrespective of past experimental treatments of heat and fish presence. New fish category includes aggregating fish (*D. aruanus*, *D. reticulatus*, *P. ambionensis*, and *P. moluccensis)* present during multiple observations. No fish SE = 0.0170, and Any fish SE: 0.0087. (b) F_V_/F_M_ of coral colonies under category of only *D. aruanus* still present. *D. aruanus* absent SE = 0.0099, and *D. aruanus* present SE=0.0126. (*) denotes a significant difference between fish treatments and *error bars* show SE. One-way analysis of variance (ANOVA) were performed on PAM data, 6-months post-experiment test for differences in F_V_/F_M_ levels in field samples of *P. damicornis*. Data for F_V_/F_M_ analysis met assumptions of normality (Shapiro-Wilks test) and homogeneity of variance (Barlett’s test).
