# Supplementary material for: Coral-dwelling fish moderate bleaching susceptibility of coral hosts
Source: PLoS One. 2018 Dec 14;13(12):e0208545. doi: 10.1371/journal.pone.0208545 (PMC6294555; doi:10.1371/journal.pone.0208545)
Supplement: S3 Fig — Photosynthetic efficiency (FV/FM) of P. damicornis colonies was recorded during: Acclimation (day 5), Stress (day 37) and Recovery (Day 66), in aquaria experiment. Linear regression analysis (Pearson’s correlation r2 = 0.5468, F1,10 = 12.07, p = 0.0060, y = 0.2266x + 0.378) suggests direct correlation between Symbiodinium and photosynthetic efficiency in experimental corals. (DOCX) [file pone.0208545.s014.docx]

**S3 Fig:** Relationship between symbionts (*Symbiodinium* density x10^6^ / cm^2^) and photosynthetic efficiency (F_V_/F_M_) of *P. damicornis* colonies at three different time periods in aquaria experiment.

*The following supplement accompanies the article*

Coral-dwelling fish moderate bleaching susceptibility of coral hosts

**List of authors**

TJ Chase^1,2^*, MS Pratchett^2^, GE Frank^1^, and MO Hoogenboom^1, 2^

___________________________________________________________________________

**S3 Fig.** Relationship between symbionts (*Symbiodinium* density x10^6^ cm^-2^) and photosynthetic efficiency (F_V_/F_M_) of *P. damicornis* colonies at three different time periods: Acclimation (day 5), Stress (day 37) and Recovery (Day 66), in aquaria experiment. Linear regression analysis (Pearson’s correlation r^2^=0.5468, F_1,10_=12.07, p=0.0060, y=0.2266x + 0.378) suggests direct correlation between *Symbiodinium* and photosynthetic efficiency in experimental corals.
